# A Kaleidoscope of Keratin Gene Expression and the Mosaic of Its Regulatory Mechanisms

**DOI:** 10.3390/ijms24065603

**Published:** 2023-03-15

**Authors:** Ekaterina P. Kalabusheva, Anastasia S. Shtompel, Alexandra L. Rippa, Sergey V. Ulianov, Sergey V. Razin, Ekaterina A. Vorotelyak

**Affiliations:** 1 Cell Biology Laboratory, Koltzov Institute of Developmental Biology, Russian Academy of Sciences, 119334 Moscow, Russia; 2 Department of Molecular Biology, Faculty of Biology, M.V. Lomonosov Moscow State University, 119234 Moscow, Russia; 3 Laboratory of Structural-Functional Organization of Chromosomes, Institute of Gene Biology, Russian Academy of Sciences, 119334 Moscow, Russia

**Keywords:** keratin genes, epithelium, enhancer, transcription regulation, chromatin spatial organization

## Abstract

Keratins are a family of intermediate filament-forming proteins highly specific to epithelial cells. A combination of expressed keratin genes is a defining property of the epithelium belonging to a certain type, organ/tissue, cell differentiation potential, and at normal or pathological conditions. In a variety of processes such as differentiation and maturation, as well as during acute or chronic injury and malignant transformation, keratin expression undergoes switching: an initial keratin profile changes accordingly to changed cell functions and location within a tissue as well as other parameters of cellular phenotype and physiology. Tight control of keratin expression implies the presence of complex regulatory landscapes within the keratin gene loci. Here, we highlight patterns of keratin expression in different biological conditions and summarize disparate data on mechanisms controlling keratin expression at the level of genomic regulatory elements, transcription factors (TFs), and chromatin spatial structure.

## 1. Introduction

The human genome contains a total of 54 functional keratin genes that code 28 keratins of type I and 26 keratins of type II. Type I keratins include 17 epithelial (K9-28) and 11 hair-specific keratins (K31-40), and type II keratins include twenty epithelial (K1-8 and K71-80) and six hair-specific keratins (K81-86) [1]. Keratin filaments are obligate heterodimers of type I and type II chains in a 1:1 molar ratio. Usually, keratin expression is described as paired due to the conserved composition of keratin heterodimers: 8/18, 5/14, 4/13, 1/10, etc.

The structure and functions of an epithelium depend on its mechanical barrier properties. The type of epithelium gradually changes from a simple single layer in the internal organ epithelia to a keratinized stratified epithelium in the skin epidermis. The keratin expression profile changes respectively: keratins 8/18, 7, 19, and 20 are typical for simple epithelium; expression of keratins 5/14 and 15 arises in pseudostratified epithelium; and stratified epithelia are positive for keratins 4/13 in mucous, keratins 1/10, 2 and 9 in the skin epidermis, and keratins 3/12 in the cornea. The widest spectrum of keratin expression is inherent to the hair follicle and the nail bed. The expression pattern of keratin has been well described and characterized [2,3,4]. Dysfunctions of tissues and organs due to violations of keratin distribution could be associated with impaired control of the keratin expression or genetic abnormalities. Since the expression pattern of keratins is very similar between humans and mice, a lot of human diseases have been modeled using mice knockouts. However, it should be taken into account that mice lack several orthologs of human keratins, and the expression pattern is also slightly different for several keratins [5].

**Figure 1:**
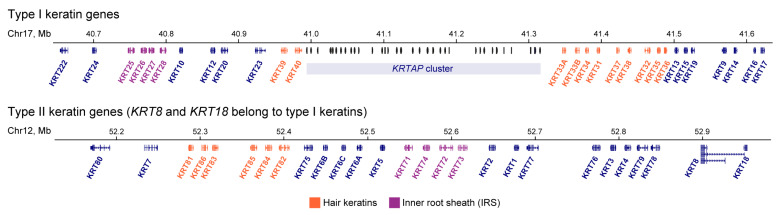
Loci of human keratin genes. Adopted from the UCSC genome browser. KRTAP—an array of genes encoding keratin-associated proteins.

Genes encoding human type I and II keratins are clustered into two distinct chromosomal regions:17q12–q21 for type I keratins (except K18) and 12q11–q13 for all type II keratins and K18 (Figure 1) [2]. The genes of conserved keratin pairs are located in different chromosomes, except keratins 8/18. Clustered localization is a conserved feature of keratin genes in terrestrial vertebrates [6] and suggests the presence of mechanisms for the coordinated keratin gene expression within clusters and on different chromosomes. In higher eukaryotes, gathering paralogous genes into a common cluster usually reflects the necessity to choose one or several of these genes to be expressed whereas keeping the other genes silent [7]. As shown in studies of several multigenic loci, i.e., protocadherin, immunoglobulin, Hox, and globin gene clusters, reconfiguration of spatial regulatory landscape within gene loci is required for the proper regulation of gene activity during differentiation and development [8,9,10,11]. In this review, we summarize the present data on the keratin gene-related transcription factors (TFs) and keratin-specific enhancers regulating keratin transcription and its switching in different biological conditions. In particular, we discuss a possible impact of a large-scale chromatin spatial structure and distant contacts between keratin regulatory elements in coordination with keratin expression.

## 2. Keratin Expression Pattern

The principal characteristic of tissue-specific keratin expression pattern is the transcription of conserved keratin pairs: keratin pair 8/18 is typical for simple epithelia, 5/14—for basal layer in pseudostratified and stratified epithelia, 6/16—for regenerating tissue, etc. However, the veritable paired expression occurs quite rarely. More often, the expression pattern of one of the paired keratins is much more widespread than that of its heterodimer partner [12,13,14]. Even when both keratin heterodimers are present in the same cell, the expression at the mRNA level may have a more significant correlation with additional keratin rather than paired keratin [15]. It is thus more correct to describe the cell type- and tissue-specific patterns of keratins as a complex consisting of a key pair of keratins and additional keratins (Table 1).

Simple epithelia keratin complex includes the keratin pair 8/18 and could be complemented with additional keratins 7, 19, 20, and 23. The first keratin 8/18 filaments are detected in a subset of cells of the eight-cell mouse embryo; their expression contributes to the isolation of the trophectoderm layer [16]. The blastocyst stage keratin expression pattern is completed by keratins 7 and 19 [145], whereas the inner cell mass is devoid of keratins [16]. In adult parenchymatous organs, the spectra of additional keratins become more complex along with an increase in the mechanical stress within an epithelium. For instance, hepatocytes of liver parenchyma are positive only for keratins 8/18, while the bile ducts also express keratins 7 and 19 [21,22]. A selective expression of keratin 23 is detected in the mouse gallbladder and common bile duct [23]. The expression of additional keratins appears de novo in pathological conditions [23,146]. In several cases, the keratin complex could lack the expression of paired keratins 8/18. Endothelia of normal human veins, venules, and lymphatics commonly exhibited focal positivity for keratin 7 and 18, whereas keratin 8 was not detected in non-neoplastic endothelia [46,47], however, most often, the loss of keratin expression indicates an epithelial-mesenchymal transition during carcinogenesis [147]. Keratins of simple epithelia are also expressed in non-epithelial tissues: smooth [47,148], skeletal [149,150], and cardiac muscle [151].

Pseudostratified epithelium combines the features of both simple and stratified-epithelia. A pair of keratins 8/18 (typical for simple epithelia) is expressed throughout the entire bulk of the pseudostratified epithelium or tends to shift to the outer layers [49,59,64]. The first keratins typical for the stratified epithelium appear in the basal layer. This probably provides increased mechanical strength of a cell mass as compared to simple epithelia. Keratin 5 is identified in the basal layer [43,50,59,64], while only a minor cell subpopulation is positive for its typical counterpart, keratin 14. Keratin 14 expression increases during injury-induced regeneration [13,51,152]. The keratins of pseudostratified epithelia could include keratins typical for other keratin complexes such as keratin 4/13 of suprabasal layers of stratified epithelia or stress-induced keratins [59].

Stratified epithelia are characterized by two complexes of keratins. Basal layers are positive for keratins 5/14 pair and additional keratin 15 [70,95,153]. The second keratin complex is expressed in the differentiating post-mitotic layers and differs depending on the epithelial localization. Mucosal epithelia express keratin pair 4/13 [154,155], cornifying epithelia are positive for keratins 1/10 with additional keratins 2 and 9 [15], keratin 12 and 3 (the last is human-specific in this complex) are expressed in specialized epithelial cells of the cornea [84].

Wound healing or other pathological conditions activate the expression of the stress-induced complex: keratins 6A, B, and C form the pair with keratins 16 and 17 [95]. These keratins promote cell migration, proliferation, and survival [156,157,158]. The stress-induced complex of keratins is associated with the downregulation of keratin 15 [159,160], keratins 1/10 [161,162], and keratins 4/13 [159], thereby, epithelia-specific keratins are replaced by the stress-induced keratins. Several wound-healing keratins expressed in healthy tissues are related to high proliferation and migration levels in hair follicles [118] and nails [129,163], or high mechanical stress in palmoplantar skin [164].

**Figure 2:**
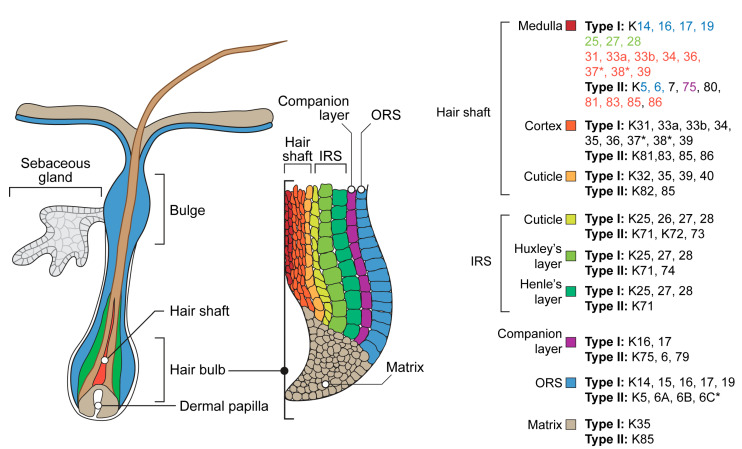
Schematic representation of the hair follicle and a list of keratins expressed in its internal structures. *—absent in the mouse genome. ORS—outer root sheath, IRS—inner root sheath. Note that keratins expressed in the medulla are colored according to their presence in other structures of the hair follicle [2,153,168,182,183,184].

The hair keratin complex possesses the widest spectrum and the most restricted localization (Figure 2). Outer root sheath (ORS), the outermost compartment which is continuous with the interfollicular epidermis, expresses keratins 5/14, 15, 6/16, and 17 [165]. A specialized part of the follicle ORS known as the bulge contains hair follicle stem cells [166,167]. During the active growth phase, anagen, bulge cells proliferate, producing progenitor cells migrating down the hair bulb at the base of the hair follicle, where they actively proliferate and generate the hair matrix. Matrix cells move upward and differentiate, developing into an inner root sheath (IRS) consisting of Henle, Huxley, and cuticle layers, as well as a hair shaft consisting of cuticle, cortex, and medulla. The keratins expressed in the IRS include 25–28 of type I and 71–74 of type II. For the hair shaft, 31–40 of type I and 81–86 of type II are typical [2,168,169]. A companion layer derived from matrix cells separates the IRS and hair shaft from the ORS. It expresses non-hair keratins: 6,16, 17, and 75 [170]. The hair medulla exists only in certain types of hair follicles: terminal in humans and guard in mice, respectively. This hair shaft central part possesses the most complicated spectra of keratins, including keratins of ORS, IRS, hair shaft cuticle, and cortex (Figure 2) [168]. Several noncanonical skin or hair keratins could be identified in hair follicles of different mammalian taxons, in particular, including a unique mammal-specific splice variant of keratin 80 in humans [171] or mucosa-related keratin 4 and 13 in cashmere goats [172]. Hair and nails, as well as other hard keratinized mammalian skin derivatives such as horns and hoofs, are examples of highly restricted unique keratin expression areas. Equine hoof lamellae epithelia, which are homologous of the nail bed, possess the expression of unique keratins 42 and 124. Murine keratin 42 expression has been localized to the nail matrix and nail bed, while the rodent orthologs of keratin 124, keratin 90, have not been characterized beyond genomic mapping and identification as likely functional genes in those species. In the human genome, K42 and K124 exist only as pseudogenes [173].

Gene targeting in a mouse model indicates the leading role of type II keratins in the organization of the above-mentioned complexes. During mouse embryogenesis, the expression of type II keratins usually precedes the expression of their type I counterparts [145]. Deletion of the entire keratin type II locus leads to the prenatal death of mouse embryos at E9.5 caused by a sequence of keratin-dependent mechanisms that leads to metabolic failure in embryonic and extraembryonic tissues [174]. Keratins 8 and 18 are first expressed in embryogenesis. Type II keratin 8 knockout results in lethality at E12.5 and complete embryos resorption at E15.5 [175], while type I keratin 18 null mice show no fetal lethality and have a normal lifespan with only minor liver abnormalities due to replacement by another type I keratin 19 [176]. Keratin 8 knockout suppresses the production of keratins 18, 19, and 20 at the protein level but not at the RNA level in the pancreas and intestines [28,177,178]. Although the expression of keratin 7 decreased, its level was still sufficient for the formation of a low amount of 7/18 and 7/19 keratin heterodimers [177]. Keratin 5 knockout induces neonatal death, while the lack of keratin 14 compensates for keratin 15 in the skin epidermis [179,180]. The importance of type II keratins is confirmed by the fact that keratin 5 is often present in the basal layer of pseudostratified epithelium, while keratin-14 expression is sparse [43,50,52,59,64]. However, recently published details about mouse epidermal keratinocytes differentiation revealed the expression of keratin 10 belonging to type I of keratins prior to type II keratin 1 at a protein level [181]. These novel results indicate the importance of further investigation of the transcription activation of paired keratins within the keratin loci for the identification of the basic principles of their coordinated expression.

## 3. Regulation of Keratin Gene Transcription

In higher eukaryotes, enhancer elements containing clustered binding sites for TFs are the main regulators of tissue-specific gene expression. Within the keratin gene loci, early studies were aimed at detecting local regulatory elements that provide effective activation of keratin gene expression. Two DNase I hypersensitive sites (HSII and HSIII) located upstream of the transcription start site (TSS) of the K14 gene were found to be jointly able to efficiently activate tissue-specific transcription of the reporter gene in transfected primary keratinocytes and transgenic mice [185,186]. Interestingly, each of these HSs alone was unable to drive tissue-specific gene expression in transgenic mice: HSII weakly activated transcription of a reporter gene in epidermal and mesenchymal cells [186], whereas HSIII drove expression in IRS cells where the K14 gene is normally inactive. This finding suggests cooperativity in the functioning of these HSs as regulators of K14 expression [187].

Similar studies using reporter constructs were carried out for other keratin genes. Enhancer elements were detected upstream from the K5 gene TSS [188] and the K6a gene [189] and within the 3′-flanking region of the K1 gene [190]. The enhancer of the K19 gene was found at a significant distance downstream from the TSS, indicating DNA looping as a potential mechanism of the enhancer-promoter contact [191]. A noteworthy example of the gene regulatory landscape is described for the K18 gene. Initially, it has been shown that a 10 kb fragment including the K18 gene is sufficient to drive tissue-specific transcription indicating that it contains all necessary regulatory information. Later, 5′- and 3′-flanking regions of this 10 kb fragment were found to ensure copy number-dependent and position-independent transcription via insulating the K18 gene from the local neighborhood [192]. Furthermore, several local enhancers have been found in this locus, including the major enhancer element located in the K18 first intron and capable of driving transcription solely [193]. This enhancer contains binding sites for AP1 and Ets TFs, jointly promoting transcription activation of the K18 gene during tumorigenesis and differentiation [194,195]. Interestingly, the first intron enhancer, minimal promoter, and 5′-flanking region of the K18 gene were used in the expression cassette to drive tissue-specific expression of the CFTR gene. This expression cassette can potentially be used for the development of gene therapy strategies for cystic fibrosis [54]. Local enhancers were also found upstream of the TSS and within the sixth exon of the K18 gene [27,196]. Similarly, local control elements are located inside the K8 coding region [197]. This complex regulatory landscape is potentially important for the K8/18 regulation during early development since this pair represents the first keratins expressed in an embryo.

The expression of several keratin genes in each epithelial cell type suggests the presence of mechanisms ensuring coordinated regulation of transcription activation/switching in keratin gene loci. One possibility is regulation by a limited set of tissue-specific and common TFs cooperatively binding to keratin gene promoters and distant regulatory regions. While a number of TFs were identified as keratin regulators in studies of individual keratin genes (Table 1), only a few TFs play the role of master-regulators of keratin loci. Specifically, the p53-related protein p63 is one of the most significant TFs controlling keratin expression [198]. p63 is present in keratin 5-positive cells of lung airway epithelia [43,50,52], esophagus [70], mammary glands [64], and skin epidermis [96,97,199]. The p63 gene is transcribed from two alternative promoters, generating several isoforms of TAp63 transcripts from an upstream promoter and ΔNp63 variants from the downstream promoter located within the intron 3. The abnormal skin phenotype observed in genetically modified mice has revealed that ΔNp63 initiates the process of differentiation and maintains the proliferation of basal layer keratinocytes, while TAp63 drives the expression of proteins specific to the upper layer of the epidermis, such as Ets-1, keratins 1/10, profilaggrin, involucrin and transglutaminase type 3 and 5 [96,199]. p63 elicits chromatin remodeling and increases chromatin accessibility at epidermal enhancers [200,201,202]. Interestingly, p63 also modulates the expression of miRNAs involved in the regulation of keratin gene transcription (for the review of the miRNA role in keratin biology, see [203]).

Other master regulators of keratin expression are Kruppel-like factors, in particular, KLF4 involved in keratin expression switching during differentiation in stratified epithelia. Klf4 knockout attenuates cells differentiation as well as keratins 4/13 expression in the esophagus [71], downregulation of keratin 12 expression in the cornea [85], and impaired skin barrier formation [98], which is also associated with declining expression of late epidermal differentiation proteins filaggrin and loricrin [99]. KLF4 and KLF5 play key roles in the proliferation and differentiation of esophagus epithelia. Klf5 maintains the low-differentiated state of epithelial cells, while Klf4 inhibits the Klf5 in progenitor cells to induce differentiation [29,71]. Microarray analysis has shown that many keratin genes were upregulated after KLF4 induction, indicating its role in epithelial differentiation [100]. Klf4 acts as a transcriptional activator of epithelial genes and as a repressor of mesenchymal genes [204] and is also able to directly bind promotor regions of several keratin genes.

Together, p63 and KLF4 are cornerstones of keratin expression regulation and keratinocyte phenotype determination since their ectopic expression induces the conversion of fibroblasts into keratinocyte-like cells by activating genes crucial for epithelial lineage specification [101,198].

In certain conditions, the expression of keratins typical for differentiated cells is repressed or markedly modulated. During wound healing, SNAI2 serves as a negative regulator of differentiation-related keratins, which probably promotes the activation of stress-induced keratins. SNAI2 is a C2H2-type zinc finger TF involved in epithelial-mesenchymal transition. Ectopic expression of SNAI2 downregulates keratin 10 as well as other differentiation markers, such as keratin 1, filaggrin, transglutaminase 1, SPRR1A, GRHL3, and KLF4 [205]. Although the expression of keratins 6, 10, and 14 are not altered in Snai2-deficient mice, the expression of keratin 8 is upregulated [206]. SNAI2 also inhibits the keratin 8/18 expression in breast cancer cells via binding to their promoter regions [207].

The hair follicle is characterized by the widest pattern of keratin expression (Table 1). The development and regeneration of the hair follicle are controlled by WNT, TGFβ, BMP, SHH, NOTCH, Eda/EdaR, etc. [208]. Lef1, a target of the WNT/β-catenin pathway, binds cooperatively with TFs Sp1, AP2-like, and NF1-like to activate hair follicle-specific genes, in particular, keratins [119]. Lef1 knockdown leads to sparse hair, with a complete loss of mouse whisker follicles [209]. Lef1 transactivates the expression of hair-specific keratins by interaction with β-catenin and presumably some other factors involved in promoting the hair cell fate [120]. Transient β-catenin stabilization may be a key event in the long-sought epidermal signal leading to hair development and implicating aberrant β-catenin activation in hair tumors [121]. WNT/β-catenin/Lef1 pathway activation is certainly one of the stages of reprogramming for interfollicular epidermal keratinocytes in the follicular direction during co-cultivation with trichogenic mesenchyme [210].

While some TFs are important for chromatin remodeling at promoters and activation of enhancers, other TFs potentially serve as looping factors mediating spatial interactions between regulatory regions within keratin loci. For instance, KLF4 forms molecular condensates and thus is able to stabilize enhancer-promoter interactions [211]. p63, which extensively binds both keratin promoters and enhancers, is also involved in the stabilization of looping interactions in cooperation with the major architectural protein CTCF [212]. Thus, the spatial structure of chromatin should be considered as another level of regulation of keratin gene expression.

## 4. Chromatin Spatial Organization as a Regulator of Keratin Gene Expression

**Figure 3:**
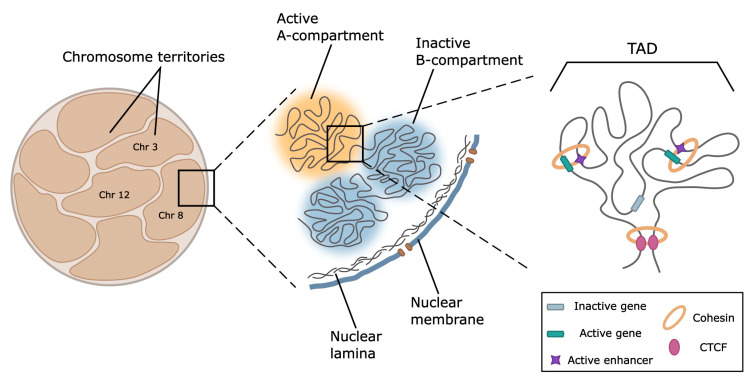
Schematic representation of the major levels of 3D genome organization. At the level of the whole nucleus and within chromosome territories, active and repressed genome loci are spatially segregated within A- and B-compartments. These structures are formed by large-distant contacts between regions possessing similar profiles of epigenetic marks. Regions forming inactive compartments tend to locate at the nucleus periphery, where they establish contact with the nuclear lamina. At the level of 100–1000 kb, chromosomes are folded into globular topologically associated domains (TADs). In mammals, TADs are predominantly formed by cohesin-driven chromatin extrusion, frequently terminating at CTCF-binding sites. In a number of well-characterized genome loci, TAD boundaries restrict the areas of enhancer action allowing regulatory systems to activate genes located within a TAD. According to the current paradigm, activation is provided by loop formation between the enhancer and cognate promoter.

Prior to the discussion of the spatial structure of keratin loci, we briefly highlight key features of the genome spatial organization (Figure 3). In interphase, chromosomes occupy discrete non-random territories in nuclear space [213]. The specific feature of chromosome territories is a high frequency of intrachromosomal contacts compared to interchromosomal contacts, as has been shown both by chromosome conformation capture-based and FISH methods [214]. However, at least in the case of erythroid-specific genes [215] and clusters of olfactory receptor genes [216], and possibly in some other loci, the interchromosomal contacts are also functionally relevant.

Within chromosome territory, active and repressed loci are segregated into A and B compartments [214], which are subdivided into a spectrum of subcompartments distinguished by the patterns of histone marks, transcription activity, and replication timing. The results of recent studies suggested phase separation as a potential mechanism of chromatin compartmentalization [217]. A number of specific factors, including the master regulator of epidermal differentiation KLF4 [211], heterochromatin-associated protein HP1α, and components of transcription machinery [218], are involved in this process. However, other results suggest that chromatin masses are solid rather than liquid [219].

At the scale of 0.1–1 Mb, the genome is partitioned into topologically associated domains (TADs) [220,221] characterized by a high frequency of contacts between loci within the domain and depletion of interactions with the neighborhood. In mammals, TADs boundaries are demarcated by the presence of CCCTC-binding factor (CTCF) and cohesin complexes playing a crucial role in the loop extrusion serving as a mechanism of TADs and loop formation [220,222]. The loop extrusion model of TADs formation suggests that cohesin progressively extrudes chromatin fiber until it encounters the barrier, such as CTCF in convergent orientation [223] or the countermovement of transcription machinery that blocks cohesin traveling [222]. The importance of these factors is shown in numerous experiments using auxin-inducible degradation of CTCF [224] and components of the cohesin complex [225]. The majority of TAD boundaries are stable across different cell types and evolutionary conserved in related species [226], as well as coincide with replication domains [227]. TAD boundary disruption [228] and CRISPR/Cas9-mediated inversion [229] result in a reconfiguration of chromatin loop topology between enhancers and promoters and lead to gene misregulation. Thus, TADs could be considered structural and functional units of the genome.

Regulatory chromatin networks at various scales significantly change during cellular differentiation and the switching of gene expression programs. Chromosomes 12 and 17, which contain clusters of the keratin genes, extensively interact with each other at an early stage of keratinocyte differentiation [230]. This is potentially important for the coordinated expression of keratin genes from the two clusters to allow for the assembly of obligate heterodimers. In another study, global screening of regulatory regions at different stages of keratinocyte differentiation showed that the sets of enhancers and superenhancers are different and specific for a particular stage of differentiation [231]. In addition, the expression of keratin genes located on chromosome 12 and a group of epithelial-specific genes was found to be significantly upregulated by induced expression of KLF4 in human cells. Coregulation of keratin genes increases the possibility of the existence of a locus control region controlled by KLF4 [100].

Dynamics of the enhancer-promoter interactions within the keratin gene locus on chromosome 12 were revealed using the Capture-C method allowing the investigation of spatial contacts between specific regions of the genome [232]. Two classes of contacts between keratin promoters and enhancers were found: static, formed in progenitor cells, and dynamic, established during cell differentiation. Dynamic contacts are characterized by the acquisition of an active H3K27ac mark during differentiation and a significant increase in contact strength, whereas pre-established (static) contacts are associated with premarked H3K27ac. Remarkably, each sort of contact is regulated by a certain set of TFs. Stable contacts are preferably associated with cohesin complex and EHF. In contrast, dynamic interactions are depleted with cohesin and require KLF4 and ZNF750, which contribute to both enhancer activation and enhancer–promoter interactions. These contacts are established within several TADs whose boundaries are stable during epidermal differentiation. Similarly, the murine epidermal differentiation complex (EDC) locus, containing genes involved in the control of terminal epidermal differentiation, consists of several gene-rich and gene-poor TADs with stable boundaries [233]. However, in contrast to the keratin gene locus, a high frequency of enhancer-promoter contacts is observed not only within the encompassing TADs but also between gene-rich TADs. In this case, a putative superenhancer in one TAD activates target genes in the other TADs illustrating that, in at least some genome loci, TAD boundaries insulate large chromosome segments from each other but cannot prevent distant regulatory interactions. Recently, it has been shown that such distant contacts within mouse keratin locus are regulated by DNA deoxygenases Tet2/3 in a DNA methylation-dependent and DNA methylation-independent manner [234]. These data are of particular interest because they raise questions about the potential modulation of keratin gene expression by artificial recruitment of DNA-methyltransferases [235].

Finally, there is a noteworthy example of the role of the 3D genome organization in providing a regional specification of the skin appendages of birds. In addition to conventional α-keratin type I and II, birds and reptiles have a special class of intermediate filament proteins (β-keratins or corneous β-proteins, while structurally these proteins are not related to keratins), which serve as “building blocks” of various skin derivatives. Transcription activation of a proper type of the β-keratin genes is provided by two different epigenetic strategies. The first strategy is a differential activation of a specific subcluster of the β-keratin genes on chromosome 25 (e.g., feather keratin genes) regulated by a superenhancer, thereby providing regional specification and development of different skin appendages (scales, claws, feathers, etc.). Another strategy is the activation of a subset of individual keratin genes required for the formation of a particular type of feather. Specificity of interaction between H3K27ac-marked regions operating as loop anchors is mediated by TFs such as KLF4 and CTCF to create the region-specific interaction networks. Moreover, genes located in the same loop are coexpressed in the same skin region or development stage shown by immunocytochemical staining. Hence, while subclusters of keratin genes on chromosome 25 are regulated as single units, differential activation of a subset of feather keratin genes on chromosome 27 occurs by changing higher-order looping configurations [236].

## 5. Conclusions and Outlooks

Clustered localization of paralogous genes is a conserved feature of vertebrate genomes. Genes encoding keratins, as well as globins, protocadherins, immunoglobulins, olfactory receptors, KRAB-ZNF TFs, and ribonucleases of the A superfamily, are grouped into relatively compact genomic domains. These loci possess certain common properties, such as preferentially unidirectional transcription, activation, and repression of individual genes in different biological conditions and cell types, and the presence of complex regulatory elements [7]. Clustered localization implies tight control of the regulatory system within a domain: activation and repression on the principle of “all or nothing” is impossible, and thus “simple solutions” such as changes in the spectrum of expressed TFs are not applicable. Precise control of promoter activity could be implemented by reconfiguration of the locus spatial structure, which, in turn, requires modulation of the regulatory and architectural elements by tuning their epigenetic state with an impact from the master-regulators of keratin complexes. Hence, we suggest the following key questions to be addressed in future studies of keratin gene loci:

(i) Reconstruction of the molecular cascades activating/switching keratin expression: from the incoming extracellular signal to activation of distinct keratin promoters.

The control of keratin expression provided by external signals from the epithelial microenvironment determines the cell stemness, the cell fate, the activation of regenerating behavior, and the regional specification of the epithelium. The plasticity of mammalian epithelia allows it to respond to stimuli from stromal and other niche cells by switching the differentiation program. Thus, epidermal keratinocytes are able to recapitulate the hair follicle [237,238] or palmo-plantar [239] skin differentiation program as well as corneal [240] and urethral [241] in tissue-specific substitutes with activation of specific keratin expression. At the same time, the key signals of newly formed epithelia-mesenchymal interactions remain unknown, as well as the intracellular pathways controlling the cell transdifferentiation, including the keratin complexes switching.

(ii) Disclosure of mechanisms preventing aberrant activation of keratin genes within complex regulatory landscapes of the keratin gene loci.

One of the markers of cancer progression and other pathologies is the appearance or upregulation of several keratin genes, including K17, K19, K6/16, etc. Keratins are not only routinely used for cancer diagnostics, but are also applicable for the prediction of invasiveness and aggressiveness of the tumor cell [242,243,244,245,246,247,248,249,250,251,252,253,254,255,256,257,258,259]. Following the aberrant expression, the reorganization of the keratin cytoskeleton affects the cell motility, survival, and resistance to external factors, which results in tumor treatment responsiveness [260,261,262,263,264,265,266,267,268,269]. The discovery of the molecular basis of aberrant keratin expression induction is one of the directions in cancer research. A potential role of oncogenetic TFs in the direct activation of individual keratin genes or possible reorganization of spatial contacts between keratin gene promoters and enhancers within keratin loci are questions to be addressed in future research.

(iii) Identification of mutations in regulatory regions associated with diseases caused by aberrant keratin expression.

Keratin gene mutations cause various hereditary diseases and keratinopathies, resulting in cellular and tissue defects [270,271,272,273,274,275,276,277,278]. Several mutations in different keratin genes manifest in very similar symptoms, thus causing different forms of the same disease. Epidermolysis bullosa simplex is a group of inherited disorders caused by mutations in at least seven different genes, including K5 and K14, characterized by recurrent blister formation as the result of skin and mucosa fragility [276,277,279,280]. Pachyonychia congenita, which could be a result of mutations in K6a, K6b, K6c, K16, or K17 genes, is associated with hypertrophic nail dystrophy and palmoplantar keratoderma [281,282]. Epidermolytic ichthyosis is caused by mutations in the genes K1, K2, and K10 [283,284,285]. Simple epithelia keratin mutations have been identified as risk factors for some intestine and liver diseases [12,286]. Many of these conditions can be reproduced for pathogenesis studies by genetically modifying mice [287]. The discovery of the genetic basis of keratin-associated disorders provides the basis for the development of reliable gene therapy strategies [288,289]. Recent evidence on the etiology and pathophysiology of inherited diseases revealed that not only mutations located in gene bodies but also in noncoding DNA could result in phenotypic abnormalities due to transcriptions disturbance [290,291,292,293,294,295,296,297]. Identification of the regulatory elements in keratin loci will provide new insights into congenital diseases driven by keratin cytoskeleton defects.

## Figures and Tables

**Table 1:** The pattern of keratin expression and TFss involved in keratin expression regulation. *—absent in the mouse genome. ORS—outer root sheath, IRS—inner root sheath.

Tissues (Organs) and Keratins	Transcription Factors	Links
**Simple Epithelia Keratin Complex**		
**Trophectoderm:** *K*8/18, 7, 19	**CDX2, TEAD4, BAF155, AP-1, Ets**	[16,17,18,19,20]
**Liver:** *Precursors: K*8/18, 19 *Hepatocytes: K*8/18 *Bile ducts: K*8/18, 7, 19, 23	**HNF-1, HNF-4alpha, HNF-3beta, C/EBPalpha, AP-1, SP1**	[21,22,23,24,25,26,27]
**Pancreas:** *Acinar cells: K*8/18, 19, 20 *Ducts: K*8/18, 7, 19, 20	**KLF4**, **KLF5**, **PDX1, MEIS1a, PBX1b, ELF3, Sp1, AP-2**	[28,29,30,31,32,33]
**Intestine:** *K*8/18, 7, 19, 20, 21	**CDX1, HNF-1α, HNF-4α, Cdx2, GATA-4** **AP1, SATB2**	[12,27,34,35,36,37,38,39,40]
**Kidney (Nephron):** *K*8/18, 7, 19	**HNF-4α, SIX1**	[36,41,42]
**Lungs (alveoli):** *K*8/18, 7, 19	**Nkx2-1**	[43,44,45]
**Endothelia:** *K*18, 7	**Hey2**	[46,47,48]
**Pseudostratified Epithelia Complex**		
**Lungs:** *Bronchioles: K*8/18, 19, minor: *K*5, 6, 7, 15, 17 *Trachea & bronchi:* *K*5, 15, 8/18, 6a, 19, minor: *K*14	**p63**, **SOX2**, **SOX21**, **KLF5**, **CFTR, ESE-2** **FOXA1/A2**	[13,43,45,49,50,51,52,53,54,55,56,57,58]
**Urinary system:** *Renal pelvis: K*8/18, 7, 19 *Bladder: K*8/18, 5, 4, 7, 19, 20, 13	**p63**, **PPARγ1, GATA3, FOXA1**	[14,59,60,61,62,63]
**Mammary gland (ducts):** *K*5/14, 8/18, 19	**p63**, **ER, FOXA1, GATA3, ZNF217, C/EBPβ, SP1**	[64,65,66,67,68,69]
**Stratified Epithelia Complex**		
**Esophagus:** *Basal layer: K*5/14, 15. *Differentiated layers: K*4/13	**p63**, **SOX2**, **KLF4**, **KLF5**, **GATA4, ESE-1**	[70,71,72,73,74,75,76,77,78,79]
**Oral mucosa:** *Basal layer: K*5/14, 15. *Differentiated layers: K*4/13	**p63**, **SOX2**, **PITX1, AP-2α, c-MYC, GRHL2**	[60,80,81,82,83]
**Cornea:** *Basal layer: K*5/14, 12 *Differentiated layers: K*3*/12	**p63**, **KLF4**, **KLF6**, **PAX6, FOXC1, ESE-1, RUNX1**	[84,85,86,87,88,89,90,91,92,93,94]
**Skin epidermis:** *Basal layer: K*5/14, 15, 19 *Differentiated layers: K*1/10, 2, 9, 6 a,b,c*/16, 17	**p63**, **KLF4**, **RUNX1, RUNX3, NF-κB, AP1, AP2, SP1, C/EBP, T3R, RAR, CHOP, GR, SETX, C-MYC, STAT3, NRF2, C/EBPα**	[95,96,97,98,99,100,101,102,103,104,105,106,107,108,109,110,111,112,113,114,115,116,117]
**Hair Follicle Keratin Complex**		
**Hair follicle:** *ORS: K*5/14, 15, 6 a,b,c*/16, 17, 19 *Companion layer: K*6 a,b,c,*/16, 17, 75, 79 *IRS: K*25-28/71-74 *Hair shaft: K*31-40/81-86	**LEF1, TCF3, SP1, AP2-like, NF1-like, NF-κB, LHX2, STAT3**, **SOX9** **(ORS), HOXC13 (hair shaft), GATA3 (IRS)**	[113,118,119,120,121,122,123,124,125,126,127,128]
**Nail:***Matrix: K*5/14, 1/10, 6 a,b,c*/16, 17, 31, 34, 85, 86 *Nail bed: K*6 a,b,c*/16, 75	**GATA3, MSX2, FOXN1, PAI2**	[117,124,129,130,131,132]
**Stress-Induced Keratin Complex**		
*K*6 a,b,c*/16,17	**p53**, **STAT1, Gli1/2, Nrf, BARX2, AP1/2, NF-κB, SP1, GR, RAR, RUNX1**	[103,106,109,133,134,135,136,137,138,139,140,141,142,143,144]
Keratins: Type I, Type II	TFs: Kruppel-like, p53 family, SOX family.	

## Abbreviations

CL	Companion layer
CTCF	CCCTC-binding factor
EDC	epidermal differentiation complex
FISH	fluorescence in situ hybridization
HS	DNase I hypersensitive site
IRS	inner root sheath
KLF	Kruppel-like factors
ORS	outer root sheath
TAD	topologically associated domain
TF	transcription factor
TSS	transcription start site.

## References

[1] Schweizer J., Bowden P.E., Coulombe P.A., Langbein L., Lane E.B., Magin T.M., Maltais L., Omary M.B., Parry D.A.D., Rogers M.A. (2006). New Consensus Nomenclature for Mammalian Keratins. J. Cell Biol..

[2] Moll R., Divo M., Langbein L. (2008). The Human Keratins: Biology and Pathology. Histochem. Cell Biol..

[3] Jacob J.T., Coulombe P.A., Kwan R., Omary M.B. (2018). Types I and II Keratin Intermediate Filaments. Cold Spring Harb. Perspect. Biol..

[4] Roth W., Hatzfeld M., Friedrich M., Thiering S., Magin T.M. (2012). Keratin Function and Regulation in Tissue Homeostasis and Pathogenesis. Biomol. Concepts.

[5] Ho M., Thompson B., Fisk J.N., Nebert D.W., Bruford E.A., Vasiliou V., Bunick C.G. (2022). Update of the Keratin Gene Family: Evolution, Tissue-Specific Expression Patterns, and Relevance to Clinical Disorders. Hum. Genom..

[6] Ehrlich F., Fischer H., Langbein L., Praetzel-Wunder S., Ebner B., Figlak K., Weissenbacher A., Sipos W., Tschachler E., Eckhart L. (2019). Differential Evolution of the Epidermal Keratin Cytoskeleton in Terrestrial and Aquatic Mammals. Mol. Biol. Evol..

[7] Razin S.V., Ioudinkova E.S., Kantidze O.L., Iarovaia O.V. (2021). Co-Regulated Genes and Gene Clusters. Genes.

[8] Iarovaia O.V., Kovina A.P., Petrova N.V., Razin S.V., Ioudinkova E.S., Vassetzky Y.S., Ulianov S.V. (2018). Genetic and Epigenetic Mechanisms of β-Globin Gene Switching. Biochem. Biokhimiia.

[9] Montavon T., Soshnikova N. (2014). Hox Gene Regulation and Timing in Embryogenesis. Semin. Cell Dev. Biol..

[10] Wu Q., Jia Z. (2021). Wiring the Brain by Clustered Protocadherin Neural Codes. Neurosci. Bull..

[11] Zhang Y., Zhang X., Dai H.-Q., Hu H., Alt F.W. (2022). The Role of Chromatin Loop Extrusion in Antibody Diversification. Nat. Rev. Immunol..

[12] Mun J., Hur W., Ku N.-O. (2022). Roles of Keratins in Intestine. Int. J. Mol. Sci..

[13] Cole B.B., Smith R.W., Jenkins K.M., Graham B.B., Reynolds P.R., Reynolds S.D. (2010). Tracheal Basal Cells: A Facultative Progenitor Cell Pool. Am. J. Pathol..

[14] Li Y., Liu Y., Gao Z., Zhang L., Chen L., Wu Z., Liu Q., Wang S., Zhou N., Chai T.C. (2021). Single-Cell Transcriptomes of Mouse Bladder Urothelium Uncover Novel Cell Type Markers and Urothelial Differentiation Characteristics. Cell Prolif..

[15] Cohen E., Johnson C., Redmond C.J., Nair R.R., Coulombe P.A. (2022). Revisiting the Significance of Keratin Expression in Complex Epithelia. J. Cell Sci..

[16] Lim H.Y.G., Alvarez Y.D., Gasnier M., Wang Y., Tetlak P., Bissiere S., Wang H., Biro M., Plachta N. (2020). Keratins Are Asymmetrically Inherited Fate Determinants in the Mammalian Embryo. Nature.

[17] Assou S., Boumela I., Haouzi D., Monzo C., Dechaud H., Kadoch I.-J., Hamamah S. (2012). Transcriptome Analysis during Human Trophectoderm Specification Suggests New Roles of Metabolic and Epigenetic Genes. PLoS ONE.

[18] Meinhardt G., Haider S., Kunihs V., Saleh L., Pollheimer J., Fiala C., Hetey S., Feher Z., Szilagyi A., Than N.G. (2020). Pivotal Role of the Transcriptional Co-Activator YAP in Trophoblast Stemness of the Developing Human Placenta. Proc. Natl. Acad. Sci. USA.

[19] Nishioka N., Inoue K., Adachi K., Kiyonari H., Ota M., Ralston A., Yabuta N., Hirahara S., Stephenson R.O., Ogonuki N. (2009). The Hippo Signaling Pathway Components Lats and Yap Pattern Tead4 Activity to Distinguish Mouse Trophectoderm from Inner Cell Mass. Dev. Cell.

[20] Wu G., Gentile L., Fuchikami T., Sutter J., Psathaki K., Esteves T.C., Araúzo-Bravo M.J., Ortmeier C., Verberk G., Abe K. (2010). Initiation of Trophectoderm Lineage Specification in Mouse Embryos Is Independent of Cdx2. Dev. Camb. Engl..

[21] Bateman A.C., Hübscher S.G. (2010). Cytokeratin Expression as an Aid to Diagnosis in Medical Liver Biopsies. Histopathology.

[22] Desmet V.J., van Eyken P., Sciot R. (1990). Cytokeratins for Probing Cell Lineage Relationships in Developing Liver. Hepatology.

[23] Guldiken N., Ensari G.K., Lahiri P., Couchy G., Preisinger C., Liedtke C., Zimmermann H.W., Ziol M., Boor P., Zucman-Rossi J. (2016). Keratin 23 Is a Stress-Inducible Marker of Mouse and Human Ductular Reaction in Liver Disease. J. Hepatol..

[24] Hakoda T., Yamamoto K., Terada R., Okano N., Shimada N., Suzuki T., Mizuno M., Shiratori Y. (2003). A Crucial Role of Hepatocyte Nuclear Factor-4 Expression in the Differentiation of Human Ductular Hepatocytes. Lab. Investig. J. Tech. Methods Pathol..

[25] Hayashi Y., Wang W., Ninomiya T., Nagano H., Ohta K., Itoh H. (1999). Liver Enriched Transcription Factors and Differentiation of Hepatocellular Carcinoma. Mol. Pathol..

[26] Rhee H., Kim H.-Y., Choi J.-H., Woo H.G., Yoo J.E., Nahm J.H., Choi J.-S., Park Y.N. (2018). Keratin 19 Expression in Hepatocellular Carcinoma Is Regulated by Fibroblast-Derived HGF via a MET-ERK1/2-AP1 and SP1 Axis. Cancer Res..

[27] Rhodes K., Oshima R.G. (1998). A Regulatory Element of the Human Keratin 18 Gene with AP-1-Dependent Promoter Activity*. J. Biol. Chem..

[28] Toivola D.M., Baribault H., Magin T., Michie S.A., Omary M.B. (2000). Simple Epithelial Keratins Are Dispensable for Cytoprotection in Two Pancreatitis Models. Am. J. Physiol.-Gastrointest. Liver Physiol..

[29] Brembeck F.H., Rustgi A.K. (2000). The Tissue-Dependent Keratin 19 Gene Transcription Is Regulated by GKLF/KLF4 and Sp1*. J. Biol. Chem..

[30] Deramaudt T.B., Sachdeva M.M., Wescott M.P., Chen Y., Stoffers D.A., Rustgi A.K. (2006). The PDX1 Homeodomain Transcription Factor Negatively Regulates the Pancreatic Ductal Cell-Specific Keratin 19 Promoter*. J. Biol. Chem..

[31] Diaferia G.R., Balestrieri C., Prosperini E., Nicoli P., Spaggiari P., Zerbi A., Natoli G. (2016). Dissection of Transcriptional and Cis-Regulatory Control of Differentiation in Human Pancreatic Cancer. EMBO J..

[32] Mauda-Havakuk M., Litichever N., Chernichovski E., Nakar O., Winkler E., Mazkereth R., Orenstein A., Bar-Meir E., Ravassard P., Meivar-Levy I. (2011). Ectopic PDX-1 Expression Directly Reprograms Human Keratinocytes along Pancreatic Insulin-Producing Cells Fate. PLoS ONE.

[33] Pujal J., Huch M., José A., Abasolo I., Rodolosse A., Duch A., Sánchez-Palazón L., Smith F.J.D., McLean W.H.I., Fillat C. (2009). Keratin 7 Promoter Selectively Targets Transgene Expression to Normal and Neoplastic Pancreatic Ductal Cells in Vitro and in Vivo. FASEB J. Off. Publ. Fed. Am. Soc. Exp. Biol..

[34] Benoit Y.D., Paré F., Francoeur C., Jean D., Tremblay E., Boudreau F., Escaffit F., Beaulieu J.-F. (2010). Cooperation between HNF-1α, Cdx2, and GATA-4 in Initiating an Enterocytic Differentiation Program in a Normal Human Intestinal Epithelial Progenitor Cell Line. Am. J. Physiol.-Gastrointest. Liver Physiol..

[35] Chan C.W.M., Wong N.A., Liu Y., Bicknell D., Turley H., Hollins L., Miller C.J., Wilding J.L., Bodmer W.F. (2009). Gastrointestinal Differentiation Marker Cytokeratin 20 Is Regulated by Homeobox Gene CDX1. Proc. Natl. Acad. Sci. USA.

[36] Chen L., Luo S., Dupre A., Vasoya R.P., Parthasarathy A., Aita R., Malhotra R., Hur J., Toke N.H., Chiles E. (2021). The Nuclear Receptor HNF4 Drives a Brush Border Gene Program Conserved across Murine Intestine, Kidney, and Embryonic Yolk Sac. Nat. Commun..

[37] Hrudka J., Matěj R., Nikov A., Tomyak I., Fišerová H., Jelínková K., Waldauf P. (2022). Loss of SATB2 Expression Correlates with Cytokeratin 7 and PD-L1 Tumor Cell Positivity and Aggressiveness in Colorectal Cancer. Sci. Rep..

[38] Lee J.A., Seo M.-K., Yoo S.-Y., Cho N.-Y., Kwak Y., Lee K., Kim J.H., Kang G.H. (2022). Comprehensive Clinicopathologic, Molecular, and Immunologic Characterization of Colorectal Carcinomas with Loss of Three Intestinal Markers, CDX2, SATB2, and KRT20. Virchows Arch. Int. J. Pathol..

[39] Sree U.D., Prayaga A.K., Reddy V.V.R., Rukmanghadha N., Chowhan A.K., Phaneendra B.V. (2022). Differential Expression of CK7, CK20, CDX2 in Intestinal and Pancreatobiliary Types of Preriampullary Carcinoma. Indian J. Pathol. Microbiol..

[40] Polari L., Alam C.M., Nyström J.H., Heikkilä T., Tayyab M., Baghestani S., Toivola D.M. (2020). Keratin Intermediate Filaments in the Colon: Guardians of Epithelial Homeostasis. Int. J. Biochem. Cell Biol..

[41] Kumaran G.K., Hanukoglu I. (2020). Identification and Classification of Epithelial Cells in Nephron Segments by Actin Cytoskeleton Patterns. Febs J..

[42] Wang P., Chen Y., Yong J., Cui Y., Wang R., Wen L., Qiao J., Tang F. (2018). Dissecting the Global Dynamic Molecular Profiles of Human Fetal Kidney Development by Single-Cell RNA Sequencing. Cell Rep..

[43] Yi H., Ku N.-O. (2013). Intermediate Filaments of the Lung. Histochem. Cell Biol..

[44] Little D.R., Gerner-Mauro K.N., Flodby P., Crandall E.D., Borok Z., Akiyama H., Kimura S., Ostrin E.J., Chen J. (2019). Transcriptional Control of Lung Alveolar Type 1 Cell Development and Maintenance by NK Homeobox 2-1. Proc. Natl. Acad. Sci. USA.

[45] Strunz M., Simon L.M., Ansari M., Kathiriya J.J., Angelidis I., Mayr C.H., Tsidiridis G., Lange M., Mattner L.F., Yee M. (2020). Alveolar Regeneration through a Krt8+ Transitional Stem Cell State That Persists in Human Lung Fibrosis. Nat. Commun..

[46] Mattey D.L., Nixon N., Wynn-Jones C., Dawes P.T. (1993). Demonstration of Cytokeratin in Endothelial Cells of the Synovial Microvasculature in Situ and in Vitro. Br. J. Rheumatol..

[47] Miettinen M., Fetsch J.F. (2000). Distribution of Keratins in Normal Endothelial Cells and a Spectrum of Vascular Tumors: Implications in Tumor Diagnosis. Hum. Pathol..

[48] Chi J.-T., Chang H.Y., Haraldsen G., Jahnsen F.L., Troyanskaya O.G., Chang D.S., Wang Z., Rockson S.G., van de Rijn M., Botstein D. (2003). Endothelial Cell Diversity Revealed by Global Expression Profiling. Proc. Natl. Acad. Sci. USA.

[49] Watson J.K., Rulands S., Wilkinson A.C., Wuidart A., Ousset M., Van Keymeulen A., Göttgens B., Blanpain C., Simons B.D., Rawlins E.L. (2015). Clonal Dynamics Reveal Two Distinct Populations of Basal Cells in Slow-Turnover Airway Epithelium. Cell Rep..

[50] Rock J.R., Onaitis M.W., Rawlins E.L., Lu Y., Clark C.P., Xue Y., Randell S.H., Hogan B.L.M. (2009). Basal Cells as Stem Cells of the Mouse Trachea and Human Airway Epithelium. Proc. Natl. Acad. Sci. USA.

[51] Hong K.U., Reynolds S.D., Watkins S., Fuchs E., Stripp B.R. (2004). In Vivo Differentiation Potential of Tracheal Basal Cells: Evidence for Multipotent and Unipotent Subpopulations. Am. J. Physiol.-Lung Cell. Mol. Physiol..

[52] Schoch K.G., Lori A., Burns K.A., Eldred T., Olsen J.C., Randell S.H. (2004). A Subset of Mouse Tracheal Epithelial Basal Cells Generates Large Colonies in Vitro. Am. J. Physiol. Lung Cell. Mol. Physiol..

[53] Bischof J.M., Ott C.J., Leir S.-H., Gosalia N., Song L., London D., Furey T.S., Cotton C.U., Crawford G.E., Harris A. (2012). A Genome-Wide Analysis of Open Chromatin in Human Tracheal Epithelial Cells Reveals Novel Candidate Regulatory Elements for Lung Function. Thorax.

[54] Chow Y.-H., Plumb J., Wen Y., Steer B.M., Lu Z., Buchwald M., Hu J. (2000). Targeting Transgene Expression to Airway Epithelia and Submucosal Glands, Prominent Sites of Human CFTR Expression. Mol. Ther..

[55] Eenjes E., Buscop-van Kempen M., Boerema-de Munck A., Edel G.G., Benthem F., de Kreij-de Bruin L., Schnater M., Tibboel D., Collins J., Rottier R.J. (2021). SOX21 Modulates SOX2-Initiated Differentiation of Epithelial Cells in the Extrapulmonary Airways. eLife.

[56] Ghosh M., Brechbuhl H.M., Smith R.W., Li B., Hicks D.A., Titchner T., Runkle C.M., Reynolds S.D. (2011). Context-Dependent Differentiation of Multipotential Keratin 14–Expressing Tracheal Basal Cells. Am. J. Respir. Cell Mol. Biol..

[57] Mollaoglu G., Jones A., Wait S.J., Mukhopadhyay A., Jeong S., Arya R., Camolotto S.A., Mosbruger T.L., Stubben C.J., Conley C.J. (2018). The Lineage-Defining Transcription Factors SOX2 and NKX2-1 Determine Lung Cancer Cell Fate and Shape the Tumor Immune Microenvironment. Immunity.

[58] Paranjapye A., NandyMazumdar M., Browne J.A., Leir S.-H., Harris A. (2021). Krüppel-like Factor 5 Regulates Wound Repair and the Innate Immune Response in Human Airway Epithelial Cells. J. Biol. Chem..

[59] Alonso A., Ikinger U., Kartenbeck J. (2009). Staining Patterns of Keratins in the Human Urinary Tract. Histol. Histopathol..

[60] Hustler A., Eardley I., Hinley J., Pearson J., Wezel F., Radvanyi F., Baker S.C., Southgate J. (2018). Differential Transcription Factor Expression by Human Epithelial Cells of Buccal and Urothelial Derivation. Exp. Cell Res..

[61] Papafotiou G., Paraskevopoulou V., Vasilaki E., Kanaki Z., Paschalidis N., Klinakis A. (2016). KRT14 Marks a Subpopulation of Bladder Basal Cells with Pivotal Role in Regeneration and Tumorigenesis. Nat. Commun..

[62] Kamasako T., Kaga K., Inoue K., Hariyama M., Yamanishi T. (2022). Supervised Machine Learning Algorithm Identified KRT20, BATF and TP63 as Biologically Relevant Biomarkers for Bladder Biopsy Specimens from Interstitial Cystitis/Bladder Pain Syndrome Patients. Int. J. Urol..

[63] Guo C.C., Bondaruk J., Yao H., Wang Z., Zhang L., Lee S., Lee J.-G., Cogdell D., Zhang M., Yang G. (2020). Assessment of Luminal and Basal Phenotypes in Bladder Cancer. Sci. Rep..

[64] Boecker W., van Horn L., Stenman G., Stürken C., Schumacher U., Loening T., Liesenfeld L., Korsching E., Gläser D., Tiemann K. (2018). Spatially Correlated Phenotyping Reveals K5-Positive Luminal Progenitor Cells and P63-K5/14-Positive Stem Cell-like Cells in Human Breast Epithelium. Lab. Investig..

[65] Asselin-Labat M.-L., Sutherland K.D., Barker H., Thomas R., Shackleton M., Forrest N.C., Hartley L., Robb L., Grosveld F.G., van der Wees J. (2007). Gata-3 Is an Essential Regulator of Mammary-Gland Morphogenesis and Luminal-Cell Differentiation. Nat. Cell Biol..

[66] Chakrabarti R., Wei Y., Hwang J., Hang X., Blanco M.A., Choudhury A., Tiede B., Romano R.-A., DeCoste C., Mercatali L. (2014). ΔNp63 Promotes Stem Cell Activity in Mammary Gland Development and Basal-like Breast Cancer by Enhancing Fzd7 Expression and Wnt Signaling. Nat. Cell Biol..

[67] LaMarca H.L., Visbal A.P., Creighton C.J., Liu H., Zhang Y., Behbod F., Rosen J.M. (2010). C/EBPβ Regulates Stem Cell Activity and Specifies Luminal Cell Fate in the Mammary Gland. Stem Cells.

[68] Verma A., Singh A., Singh M.P., Nengroo M.A., Saini K.K., Satrusal S.R., Khan M.A., Chaturvedi P., Sinha A., Meena S. (2022). EZH2-H3K27me3 Mediated KRT14 Upregulation Promotes TNBC Peritoneal Metastasis. Nat. Commun..

[69] Raap M., Gierendt L., Werlein C., Kuehnle E., Kreipe H.H., Christgen M. (2021). Co-Expression of Transcription Factor AP-2beta (TFAP2B) and GATA3 in Human Mammary Epithelial Cells with Intense, Apicobasal Immunoreactivity for CK8/18. J. Mol. Histol..

[70] Giroux V., Lento A.A., Islam M., Pitarresi J.R., Kharbanda A., Hamilton K.E., Whelan K.A., Long A., Rhoades B., Tang Q. (2017). Long-Lived Keratin 15^+^ Esophageal Progenitor Cells Contribute to Homeostasis and Regeneration. J. Clin. Investig..

[71] Tetreault M.-P., Yang Y., Travis J., Yu Q.-C., Klein-Szanto A., Tobias J.W., Katz J.P. (2010). Esophageal Squamous Cell Dysplasia and Delayed Differentiation with Deletion of Klf4 in Murine Esophagus. Gastroenterology.

[72] Jenkins T.D., Opitz O.G., Okano J., Rustgi A.K. (1998). Transactivation of the Human Keratin 4 and Epstein-Barr Virus ED-L2 Promoters by Gut-Enriched Krüppel-like Factor. J. Biol. Chem..

[73] Brembeck F.H., Opitz O.G., Libermann T.A., Rustgi A.K. (2000). Dual Function of the Epithelial Specific Ets Transcription Factor, ELF3, in Modulating Differentiation. Oncogene.

[74] DeWard A.D., Cramer J., Lagasse E. (2014). Cellular Heterogeneity in the Mouse Esophagus Implicates the Presence of a Nonquiescent Epithelial Stem Cell Population. Cell Rep..

[75] Que J., Okubo T., Goldenring J.R., Nam K.-T., Kurotani R., Morrisey E.E., Taranova O., Pevny L.H., Hogan B.L.M. (2007). Multiple Dose-Dependent Roles for Sox2 in the Patterning and Differentiation of Anterior Foregut Endoderm. Dev. Camb. Engl..

[76] Stavniichuk R., DeLaForest A., Thompson C.A., Miller J., Souza R.F., Battle M.A. (2021). GATA4 Blocks Squamous Epithelial Cell Gene Expression in Human Esophageal Squamous Cells. Sci. Rep..

[77] Yu W.-Y., Slack J.M.W., Tosh D. (2005). Conversion of Columnar to Stratified Squamous Epithelium in the Developing Mouse Oesophagus. Dev. Biol..

[78] Whelan K.A., Muir A.B., Nakagawa H. (2018). Esophageal 3D Culture Systems as Modeling Tools in Esophageal Epithelial Pathobiology and Personalized Medicine. Cell. Mol. Gastroenterol. Hepatol..

[79] Sankoda N., Tanabe W., Tanaka A., Shibata H., Woltjen K., Chiba T., Haga H., Sakai Y., Mandai M., Yamamoto T. (2021). Epithelial Expression of Gata4 and Sox2 Regulates Specification of the Squamous–Columnar Junction via MAPK/ERK Signaling in Mice. Nat. Commun..

[80] Attico E., Galaverni G., Bianchi E., Losi L., Manfredini R., Lambiase A., Rama P., Pellegrini G. (2022). SOX2 Is a Univocal Marker for Human Oral Mucosa Epithelium Useful in Post-COMET Patient Characterization. Int. J. Mol. Sci..

[81] Iglesias-Bartolome R., Uchiyama A., Molinolo A.A., Abusleme L., Brooks S.R., Callejas-Valera J.L., Edwards D., Doci C., Asselin-Labat M.-L., Onaitis M.W. (2018). Transcriptional Signature Primes Human Oral Mucosa for Rapid Wound Healing. Sci. Transl. Med..

[82] Katada R., Tanaka J., Takamatsu K., Hata K., Yasuhara R., Ohnuma S., Takakura I., Nishimura R., Shirota T., Mishima K. (2022). Induction of Salivary Gland-like Cells from Epithelial Tissues Transdifferentiated from Mouse Embryonic Fibroblasts. Biochem. Biophys. Res. Commun..

[83] Ebersole J.L., Orraca L., Novak M.J., Kirakodu S., Gonzalez-Martinez J., Gonzalez O.A. (2019). Comparative Analysis of Gene Expression Patterns for Oral Epithelium-Related Functions with Aging. Adv. Exp. Med. Biol..

[84] Chaloin-Dufau C., Pavitt I., Delorme P., Dhouailly D. (1993). Identification of Keratins 3 and 12 in Corneal Epithelium of Vertebrates. Epithel. Cell Biol..

[85] Swamynathan S.K., Katz J.P., Kaestner K.H., Ashery-Padan R., Crawford M.A., Piatigorsky J. (2007). Conditional Deletion of the Mouse Klf4 Gene Results in Corneal Epithelial Fragility, Stromal Edema, and Loss of Conjunctival Goblet Cells. Mol. Cell. Biol..

[86] Chiambaretta F., Blanchon L., Rabier B., Kao W.W.-Y., Liu J.J., Dastugue B., Rigal D., Sapin V. (2002). Regulation of Corneal Keratin-12 Gene Expression by the Human Krüppel-like Transcription Factor 6. Investig. Ophthalmol. Vis. Sci..

[87] Liu J.J., Kao W.W., Wilson S.E. (1999). Corneal Epithelium-Specific Mouse Keratin K12 Promoter. Exp. Eye Res..

[88] Yoshida N., Yoshida S., Araie M., Handa H., Nabeshima Y. (2000). Ets Family Transcription Factor ESE-1 Is Expressed in Corneal Epithelial Cells and Is Involved in Their Differentiation. Mech. Dev..

[89] Lupasco T., He Z., Cassagne M., Sagnial T., Brion L., Fournié P., Gain P., Thuret G., Allouche M., Malecaze F. (2022). Corneal Epithelium in Keratoconus Underexpresses Active NRF2 and a Subset of Oxidative Stress-Related Genes. PLoS ONE.

[90] Li M., Zhu L., Liu J., Huang H., Guo H., Wang L., Li L., Gu S., Tan J., Zhong J. (2021). Loss of FOXC1 Contributes to the Corneal Epithelial Fate Switch and Pathogenesis. Signal Transduct. Target. Ther..

[91] Fujimoto S., Hayashi R., Hara S., Sasamoto Y., Harrington J., Tsujikawa M., Nishida K. (2019). KLF4 Prevents Epithelial to Mesenchymal Transition in Human Corneal Epithelial Cells via Endogenous TGF-Β2 Suppression. Regen. Ther..

[92] Guo Z.H., Zeng Y.M., Lin J.S. (2020). Dynamic Spatiotemporal Expression Pattern of Limbal Stem Cell Putative Biomarkers during Mouse Development. Exp. Eye Res..

[93] Kitazawa K., Hikichi T., Nakamura T., Nakamura M., Sotozono C., Masui S., Kinoshita S. (2019). Direct Reprogramming Into Corneal Epithelial Cells Using a Transcriptional Network Comprising PAX6, OVOL2, and KLF4. Cornea.

[94] Smits J., Lima Cunha D., Jieqiong Q., Owen N., Latta L., Szentmáry N., Seitz B., Roux L., Moosajee M., Aberdam D. (2022). Multi-Omics Analyses Identify Transcription Factor Interplay in Corneal Epithelial Fate Determination and Disease. bioRxiv.

[95] Zhang L. (2018). Keratins in Skin Epidermal Development and Diseases.

[96] Romano R.-A., Ortt K., Birkaya B., Smalley K., Sinha S. (2009). An Active Role of the ΔN Isoform of P63 in Regulating Basal Keratin Genes K5 and K14 and Directing Epidermal Cell Fate. PLoS ONE.

[97] Brauweiler A.M., Leung D.Y.M., Goleva E. (2021). The Transcription Factor P63 Is a Direct Effector of IL-4- and IL-13-Mediated Repression of Keratinocyte Differentiation. J. Investig. Dermatol..

[98] Segre J.A., Bauer C., Fuchs E. (1999). Klf4 Is a Transcription Factor Required for Establishing the Barrier Function of the Skin. Nat. Genet..

[99] Jaubert J., Cheng J., Segre J.A. (2003). Ectopic Expression of Kruppel like Factor 4 (Klf4) Accelerates Formation of the Epidermal Permeability Barrier. Development.

[100] Chen X., Whitney E.M., Gao S.Y., Yang V.W. (2003). Transcriptional Profiling of Krüppel-like Factor 4 Reveals a Function in Cell Cycle Regulation and Epithelial Differentiation. J. Mol. Biol..

[101] Chen Y., Mistry D., Sen G. (2013). Highly Rapid and Efficient Conversion of Human Fibroblasts to Keratinocyte-Like Cells. J. Investig. Dermatol..

[102] Lee D.-D., Stojadinovic O., Krzyzanowska A., Vouthounis C., Blumenberg M., Tomic-Canic M. (2009). Retinoid-Responsive Transcriptional Changes in Epidermal Keratinocytes. J. Cell. Physiol..

[103] Ma S., Rao L., Freedberg I.M., Blumenberg M. (2018). Transcriptional Control of K5, K6, K14, and K17 Keratin Genes by AP-1 and NF-ΚB Family Members. Gene Expr..

[104] Maytin E.V., Lin J.C., Krishnamurthy R., Batchvarova N., Ron D., Mitchell P.J., Habener J.F. (1999). Keratin 10 Gene Expression during Differentiation of Mouse Epidermis Requires Transcription Factors C/EBP and AP-2. Dev. Biol..

[105] Ogawa E., Edamitsu T., Ohmori H., Kohu K., Kurokawa M., Kiyonari H., Satake M., Okuyama R. (2022). Transcription Factors Runx1 and Runx3 Suppress Keratin Expression in Undifferentiated Keratinocytes. Int. J. Mol. Sci..

[106] Ohtsuki M., Flanagan S., Freedberg I.M., Blumenberg M. (2018). A Cluster of Five Nuclear Proteins Regulates Keratin Gene Transcription. Gene Expr..

[107] Radoja N., Stojadinovic O., Waseem A., Tomic-Canic M., Milisavljevic V., Teebor S., Blumenberg M. (2004). Thyroid Hormones and Gamma Interferon Specifically Increase K15 Keratin Gene Transcription. Mol. Cell. Biol..

[108] Maytin E.V., Habener J.F. (1998). Transcription Factors C/EBP Alpha, C/EBP Beta, and CHOP (Gadd153) Expressed during the Differentiation Program of Keratinocytes in Vitro and in Vivo. J. Investig. Dermatol..

[109] Radoja N., Komine M., Jho S.H., Blumenberg M., Tomic-Canic M. (2000). Novel Mechanism of Steroid Action in Skin through Glucocorticoid Receptor Monomers. Mol. Cell. Biol..

[110] Gatti V., Fierro C., Compagnone M., La Banca V., Mauriello A., Montanaro M., Scalera S., De Nicola F., Candi E., Ricci F. (2022). ΔNp63-Senataxin Circuit Controls Keratinocyte Differentiation by Promoting the Transcriptional Termination of Epidermal Genes. Proc. Natl. Acad. Sci. USA.

[111] Zhang J., Yang P., Liu D., Gao M., Wang J., Wang X., Liu Y., Zhang X. (2021). C-Myc Upregulated by High Glucose Inhibits HaCaT Differentiation by S100A6 Transcriptional Activation. Front. Endocrinol..

[112] Dai X., Shiraishi K., Muto J., Utsunomiya R., Mori H., Murakami M., Sayama K. (2022). Nuclear IL-33 Plays an Important Role in IL-31–Mediated Downregulation of FLG, Keratin 1, and Keratin 10 by Regulating Signal Transducer and Activator of Transcription 3 Activation in Human Keratinocytes. J. Investig. Dermatol..

[113] Miyauchi K., Ki S., Ukai M., Suzuki Y., Inoue K., Suda W., Matsui T., Ito Y., Honda K., Koseki H. (2021). Essential Role of STAT3 Signaling in Hair Follicle Homeostasis. Front. Immunol..

[114] Ogawa T., Ishitsuka Y., Inoue S., Nakamura Y., Saito A., Okiyama N., Fujisawa Y., Furuta J., Watanabe R., Fujimoto M. (2020). Nuclear Factor Erythroid 2-Related Factor 2 (Nrf2) Regulates Epidermal Keratinization under Psoriatic Skin Inflammation. Am. J. Pathol..

[115] Wu R., Zhang H., Zhao M., Li J., Hu Y., Fu J., Pi J., Wang H., Xu Y. (2020). Nrf2 in Keratinocytes Protects against Skin Fibrosis via Regulating Epidermal Lesion and Inflammatory Response. Biochem. Pharmacol..

[116] Zhuang L., Ma W., Yan J., Zhong H. (2020). Evaluation of the Effects of IL-22 on the Proliferation and Differentiation of Keratinocytes in Vitro. Mol. Med. Rep..

[117] Bowden P.E. (2010). Mutations in a Keratin 6 Isomer (K6c) Cause a Type of Focal Palmoplantar Keratoderma. J. Investig. Dermatol..

[118] Bernot K.M., Coulombe P.A., McGowan K.M. (2002). Keratin 16 Expression Defines a Subset of Epithelial Cells During Skin Morphogenesis and the Hair Cycle. J. Investig. Dermatol..

[119] Dunn S.M., Keough R.A., Rogers G.E., Powell B.C. (1998). Regulation of a Hair Follicle Keratin Intermediate Filament Gene Promoter. J. Cell Sci..

[120] Merrill B.J., Gat U., DasGupta R., Fuchs E. (2001). Tcf3 and Lef1 Regulate Lineage Differentiation of Multipotent Stem Cells in Skin. Genes Dev..

[121] Gat U., DasGupta R., Degenstein L., Fuchs E. (1998). De Novo Hair Follicle Morphogenesis and Hair Tumors in Mice Expressing a Truncated β-Catenin in Skin. Cell.

[122] DasGupta R., Fuchs E. (1999). Multiple Roles for Activated LEF/TCF Transcription Complexes during Hair Follicle Development and Differentiation. Development.

[123] Jave-Suarez L.F., Winter H., Langbein L., Rogers M.A., Schweizer J. (2002). HOXC13 Is Involved in the Regulation of Human Hair Keratin Gene Expression*. J. Biol. Chem..

[124] Kaufman C.K., Zhou P., Pasolli H.A., Rendl M., Bolotin D., Lim K.-C., Dai X., Alegre M.-L., Fuchs E. (2003). GATA-3: An Unexpected Regulator of Cell Lineage Determination in Skin. Genes Dev..

[125] Vidal V.P.I., Chaboissier M.-C., Lützkendorf S., Cotsarelis G., Mill P., Hui C.-C., Ortonne N., Ortonne J.-P., Schedl A. (2005). Sox9 Is Essential for Outer Root Sheath Differentiation and the Formation of the Hair Stem Cell Compartment. Curr. Biol..

[126] Cao M., Zhao J., Du L., Chen Z., Zhang L., Liu X., Cheng J., Yan Y., Zhang C., Li H. (2021). The Combination of Hair Follicle-Specific Marker LHX2 and Co-Expressed Marker Can Distinguish between Sweat Gland Placodes and Hair Placodes in Rat. J. Mol. Histol..

[127] Mokry J., Pisal R. (2020). Development and Maintenance of Epidermal Stem Cells in Skin Adnexa. Int. J. Mol. Sci..

[128] Wang J., He J., Zhu M., Han Y., Yang R., Liu H., Xu X., Chen X. (2022). Cellular Heterogeneity and Plasticity of Skin Epithelial Cells in Wound Healing and Tumorigenesis. Stem Cell Rev. Rep..

[129] Perrin C. (2007). Expression of Follicular Sheath Keratins in the Normal Nail With Special Reference to the Morphological Analysis of the Distal Nail Unit. Am. J. Dermatopathol..

[130] Cai J., Ma L. (2011). Msx2 and Foxn1 Regulate Nail Homeostasis. Genes.

[131] Liu M., Li F., Wang X., Liu Z., Wong H.S., Zhou Y., Wang D. (2022). Expression Patterns of Hair-Related Keratins and Epithelial Keratins in Onychopapilloma: The Significance of Clarifying the Origin of Onychopapilloma. Front. Med..

[132] Rice R.H., Xia Y., Alvarado R.J., Phinney B.S. Proteomic Analysis of Human Nail Plate. https://pubs.acs.org/doi/pdf/10.1021/pr1009349.

[133] Komine M., Freedberg I.M., Blumenberg M. (1996). Regulation of Epidermal Expression of Keratin K17 in Inflammatory Skin Diseases. J. Investig. Dermatol..

[134] Lu Z., Peng H., Li R., Xu X., Peng J. (2022). BarH-like Homeobox 2 Represses the Transcription of Keratin 16 and Affects Ras Signaling Pathway to Suppress Nasopharyngeal Carcinoma Progression. Bioengineered.

[135] Mikami Y., Fujii S., Nagata K., Wada H., Hasegawa K., Abe M., Yoshimoto R.U., Kawano S., Nakamura S., Kiyoshima T. (2017). GLI-Mediated Keratin 17 Expression Promotes Tumor Cell Growth through the Anti-Apoptotic Function in Oral Squamous Cell Carcinomas. J. Cancer Res. Clin. Oncol..

[136] Navarro J.M., Casatorres J., Jorcano J.L. (1995). Elements Controlling the Expression and Induction of the Skin Hyperproliferation-Associated Keratin K6 (∗). J. Biol. Chem..

[137] Wang Y.-N., Chang W.-C. (2003). Induction of Disease-Associated Keratin 16 Gene Expression by Epidermal Growth Factor Is Regulated through Cooperation of Transcription Factors Sp1 and c-Jun. J. Biol. Chem..

[138] Yang L., Fan X., Cui T., Dang E., Wang G. (2017). Nrf2 Promotes Keratinocyte Proliferation in Psoriasis through Up-Regulation of Keratin 6, Keratin 16, and Keratin 17. J. Investig. Dermatol..

[139] Zhang X., Yin M., Zhang L. (2019). Keratin 6, 16 and 17—Critical Barrier Alarmin Molecules in Skin Wounds and Psoriasis. Cells.

[140] Yang L., Zhang S., Wang G. (2019). Keratin 17 in Disease Pathogenesis: From Cancer to Dermatoses. J. Pathol..

[141] Lombardo G., Melzi G., Indino S., Piazza S., Sangiovanni E., Baruffaldi Preis F., Marabini L., Donetti E. (2022). Keratin 17 as a Marker of UVB-Induced Stress in Human Epidermis and Modulation by Vitis Vinifera Extract. Cells Tissues Organs.

[142] Cornaghi L., Gagliano N., Preis F.W.B., Prignano F., Donetti E. (2022). Inside-out and Outside-in Organotypic Normal Human Skin Culture: JAK-STAT Pathway Is Activated after pro-Inflammatory Psoriatic Cytokine Exposure. Tissue Cell.

[143] Dai X., Utsunomiya R., Shiraishi K., Mori H., Muto J., Murakami M., Sayama K. (2021). Nuclear IL-33 Plays an Important Role in the Suppression of FLG, LOR, Keratin 1, and Keratin 10 by IL-4 and IL-13 in Human Keratinocytes. J. Investig. Dermatol..

[144] Bai X., Yu C., Yang L., Luo Y., Zhi D., Wang G., Dang E. (2020). Anti-Psoriatic Properties of Paeoniflorin: Suppression of the NF-KappaB Pathway and Keratin 17. Eur. J. Dermatol. EJD.

[145] Lu H., Hesse M., Peters B., Magin T.M. (2005). Type II Keratins Precede Type I Keratins during Early Embryonic Development. Eur. J. Cell Biol..

[146] Sakellariou S., Michaelides C., Voulgaris T., Vlachogiannakos J., Manesis E., Tiniakos D.G., Delladetsima I. (2021). Keratin 7 Expression in Hepatic Cholestatic Diseases. Virchows Arch..

[147] Menz A., Weitbrecht T., Gorbokon N., Büscheck F., Luebke A.M., Kluth M., Hube-Magg C., Hinsch A., Höflmayer D., Weidemann S. (2021). Diagnostic and Prognostic Impact of Cytokeratin 18 Expression in Human Tumors: A Tissue Microarray Study on 11,952 Tumors. Mol. Med. Camb. Mass.

[148] Bader B.L., Jahn L., Franke W.W. (1988). Low Level Expression of Cytokeratins 8, 18 and 19 in Vascular Smooth Muscle Cells of Human Umbilical Cord and in Cultured Cells Derived Therefrom, with an Analysis of the Chromosomal Locus Containing the Cytokeratin 19 Gene. Eur. J. Cell Biol..

[149] Muriel J.M., O’Neill A., Kerr J.P., Kleinhans-Welte E., Lovering R.M., Bloch R.J. (2020). Keratin 18 Is an Integral Part of the Intermediate Filament Network in Murine Skeletal Muscle. Am. J. Physiol.-Cell Physiol..

[150] Shah S., Love J., O’Neill A., Lovering R., Bloch R. (2012). Influences of Desmin and Keratin 19 on Passive Biomechanical Properties of Mouse Skeletal Muscle. J. Biomed. Biotechnol..

[151] Kuruc N., Franke W.W. (1988). Transient Coexpression of Desmin and Cytokeratins 8 and 18 in Developing Myocardial Cells of Some Vertebrate Species. Differ. Res. Biol. Divers..

[152] Ievlev V., Lynch T.J., Freischlag K.W., Gries C.B., Shah A., Pai A.C., Ahlers B.A., Park S., Engelhardt J.F., Parekh K.R. (2023). Krt14 and Krt15 Differentially Regulate Regenerative Properties and Differentiation Potential of Airway Basal Cells. JCI Insight.

[153] Liu Y., Lyle S., Yang Z., Cotsarelis G. (2003). Keratin 15 Promoter Targets Putative Epithelial Stem Cells in the Hair Follicle Bulge. J. Investig. Dermatol..

[154] Barrett A.W., Selvarajah S., Franey S., Wills K.A., Berkovitz B.K. (1998). Interspecies Variations in Oral Epithelial Cytokeratin Expression. J. Anat..

[155] Tomakidi P., Breitkreutz D., Fusenig N.E., Zöller J., Kohl A., Komposch G. (1998). Establishment of Oral Mucosa Phenotype in Vitro in Correlation to Epithelial Anchorage. Cell Tissue Res..

[156] Paladini R.D., Takahashi K., Bravo N.S., Coulombe P.A. (1996). Onset of Re-Epithelialization after Skin Injury Correlates with a Reorganization of Keratin Filaments in Wound Edge Keratinocytes: Defining a Potential Role for Keratin 16. J. Cell Biol..

[157] Wang F., Chen S., Liu H.B., Parent C.A., Coulombe P.A. (2018). Keratin 6 Regulates Collective Keratinocyte Migration by Altering Cell–Cell and Cell–Matrix Adhesion. J. Cell Biol..

[158] Pang B., Zhu Z., Xiao C., Luo Y., Fang H., Bai Y., Sun Z., Ma J., Dang E., Wang G. (2021). Keratin 17 Is Required for Lipid Metabolism in Keratinocytes and Benefits Epidermal Permeability Barrier Homeostasis. Front. Cell Dev. Biol..

[159] Sakamoto K., Aragaki T., Morita K., Kawachi H., Kayamori K., Nakanishi S., Omura K., Miki Y., Okada N., Katsube K. (2011). Down-Regulation of Keratin 4 and Keratin 13 Expression in Oral Squamous Cell Carcinoma and Epithelial Dysplasia: A Clue for Histopathogenesis. Histopathology.

[160] Waseem A., Alam Y., Lalli A., Dogan B., Tidman N., Purkis P., Jackson S., Machesney M., Leigh I.M. (1999). Keratin 15 Expression in Stratified Epithelia: Downregulation in Activated Keratinocytes. J. Investig. Dermatol..

[161] Bloor B.K., Tidman N., Leigh I.M., Odell E., Dogan B., Wollina U., Ghali L., Waseem A. (2003). Expression of Keratin K2e in Cutaneous and Oral Lesions: Association with Keratinocyte Activation, Proliferation, and Keratinization. Am. J. Pathol..

[162] Totsuka A., Omori-Miyake M., Kawashima M., Yagi J., Tsunemi Y. (2017). Expression of Keratin 1, Keratin 10, Desmoglein 1 and Desmocollin 1 in the Epidermis: Possible Downregulation by Interleukin-4 and Interleukin-13 in Atopic Dermatitis. Eur. J. Dermatol. EJD.

[163] Perrin C., Langbein L., Schweizer J. (2004). Expression of Hair Keratins in the Adult Nail Unit: An Immunohistochemical Analysis of the Onychogenesis in the Proximal Nail Fold, Matrix and Nail Bed. Br. J. Dermatol..

[164] Lessard J.C., Coulombe P.A. (2012). Keratin 16-Null Mice Develop Palmoplantar Keratoderma, a Hallmark Feature of Pachyonychia Congenita and Related Disorders. J. Investig. Dermatol..

[165] Langbein L., Schweizer J. (2005). Keratins of the Human Hair Follicle11This Article Is Dedicated with Gratitude to Werner W. Franke on the Occasion of His 65th Birthday. His Pioneering Work on Epithelial and Hair Keratins Has Been Pivotal to Our Own Investigations in This Field. International Review of Cytology.

[166] Chovatiya G., Ghuwalewala S., Walter L.D., Cosgrove B.D., Tumbar T. (2021). High-Resolution Single-Cell Transcriptomics Reveals Heterogeneity of Self-Renewing Hair Follicle Stem Cells. Exp. Dermatol..

[167] Cheng X., Yu Z., Song Y., Zhang Y., Du J., Su Y., Ma X. (2020). Hair Follicle Bulge-Derived Stem Cells Promote Tissue Regeneration during Skin Expansion. Biomed. Pharmacother..

[168] Langbein L., Yoshida H., Praetzel-Wunder S., Parry D., Schweizer J. (2009). The Keratins of the Human Beard Hair Medulla: The Riddle in the Middle. J. Investig. Dermatol..

[169] Schweizer J., Langbein L., Rogers M.A., Winter H. (2007). Hair Follicle-Specific Keratins and Their Diseases. Exp. Cell Res..

[170] Winter H., Langbein L., Praetzel S., Jacobs M., Rogers M.A., Leigh I.M., Tidman N., Schweizer J. (1998). A Novel Human Type II Cytokeratin, K6hf, Specifically Expressed in the Companion Layer of the Hair Follicle. J. Investig. Dermatol..

[171] Langbein L., Eckhart L., Rogers M.A., Praetzel-Wunder S., Schweizer J. (2010). Against the Rules: Human Keratin K80: Two Functional Alternative Splice Variants, K80 and K80.1, with Special Cellular Localization in a Wide Range of Epithelia. J. Biol. Chem..

[172] Nocelli C., Cappelli K., Capomaccio S., Pascucci L., Mercati F., Pazzaglia I., Mecocci S., Antonini M., Renieri C. (2020). Shedding Light on Cashmere Goat Hair Follicle Biology: From Morphology Analyses to Transcriptomic Landascape. BMC Genom..

[173] Armstrong C., Cassimeris L., Da Silva Santos C., Micoogullari Y., Wagner B., Babasyan S., Brooks S., Galantino-Homer H. (2019). The Expression of Equine Keratins K42 and K124 Is Restricted to the Hoof Epidermal Lamellae of Equus Caballus. PLoS ONE.

[174] Kröger C., Vijayaraj P., Reuter U., Windoffer R., Simmons D., Heukamp L., Leube R., Magin T.M. (2011). Placental Vasculogenesis Is Regulated by Keratin-Mediated Hyperoxia in Murine Decidual Tissues. Am. J. Pathol..

[175] Baribault H., Price J., Miyai K., Oshima R.G. (1993). Mid-Gestational Lethality in Mice Lacking Keratin 8. Genes Dev..

[176] Magin T.M., Schröder R., Leitgeb S., Wanninger F., Zatloukal K., Grund C., Melton D.W. (1998). Lessons from Keratin 18 Knockout Mice: Formation of Novel Keratin Filaments, Secondary Loss of Keratin 7 and Accumulation of Liver-Specific Keratin 8-Positive Aggregates. J. Cell Biol..

[177] Stenvall C.-G.A., Tayyab M., Grönroos T.J., Ilomäki M.A., Viiri K., Ridge K.M., Polari L., Toivola D.M. (2021). Targeted Deletion of Keratin 8 in Intestinal Epithelial Cells Disrupts Tissue Integrity and Predisposes to Tumorigenesis in the Colon. Cell. Mol. Life Sci..

[178] Toivola D.M., Nakamichi I., Strnad P., Michie S.A., Ghori N., Harada M., Zeh K., Oshima R.G., Baribault H., Omary M.B. (2008). Keratin Overexpression Levels Correlate with the Extent of Spontaneous Pancreatic Injury. Am. J. Pathol..

[179] Lloyd C., Yu Q.C., Cheng J., Turksen K., Degenstein L., Hutton E., Fuchs E. (1995). The Basal Keratin Network of Stratified Squamous Epithelia: Defining K15 Function in the Absence of K14. J. Cell Biol..

[180] Peters B., Kirfel J., Büssow H., Vidal M., Magin T.M. (2001). Complete Cytolysis and Neonatal Lethality in Keratin 5 Knockout Mice Reveal Its Fundamental Role in Skin Integrity and in Epidermolysis Bullosa Simplex. Mol. Biol. Cell.

[181] Cockburn K., Annusver K., Gonzalez D.G., Ganesan S., May D.P., Mesa K.R., Kawaguchi K., Kasper M., Greco V. (2022). Gradual Differentiation Uncoupled from Cell Cycle Exit Generates Heterogeneity in the Epidermal Stem Cell Layer. Nat. Cell Biol..

[182] Morgan H.J., Benketah A., Olivero C., Rees E., Ziaj S., Mukhtar A., Lanfredini S., Patel G.K. (2020). Hair Follicle Differentiation-Specific Keratin Expression in Human Basal Cell Carcinoma. Clin. Exp. Dermatol..

[183] Gu L.-H., Coulombe P.A. (2007). Keratin Expression Provides Novel Insight into the Morphogenesis and Function of the Companion Layer in Hair Follicles. J. Investig. Dermatol..

[184] Mesler A.L., Veniaminova N.A., Lull M.V., Wong S.Y. (2017). Hair Follicle Terminal Differentiation Is Orchestrated by Distinct Early and Late Matrix Progenitors. Cell Rep..

[185] Leask A., Rosenberg M., Vassar R., Fuchs E. (1990). Regulation of a Human Epidermal Keratin Gene: Sequences and Nuclear Factors Involved in Keratinocyte-Specific Transcription. Genes Dev..

[186] Sinha S., Degenstein L., Copenhaver C., Fuchs E. (2000). Defining the Regulatory Factors Required for Epidermal Gene Expression. Mol. Cell. Biol..

[187] Sinha S., Fuchs E. (2001). Identification and Dissection of an Enhancer Controlling Epithelial Gene Expression in Skin. Proc. Natl. Acad. Sci. USA.

[188] Kaufman C.K., Sinha S., Bolotin D., Fan J., Fuchs E. (2002). Dissection of a Complex Enhancer Element: Maintenance of Keratinocyte Specificity but Loss of Differentiation Specificity. Mol. Cell. Biol..

[189] Takahashi K., Coulombe P.A. (1997). Defining a Region of the Human Keratin 6a Gene That Confers Inducible Expression in Stratified Epithelia of Transgenic Mice. J. Biol. Chem..

[190] Rothnagel J.A., Greenhalgh D.A., Gagne T.A., Longley M.A., Roop D.R. (1993). Identification of a Calcium-Inducible, Epidermal-Specific Regulatory Element in the 3′-Flanking Region of the Human Keratin 1 Gene. J. Investig. Dermatol..

[191] Hu L., Gudas L.J. (1994). Activation of Keratin 19 Gene Expression by a 3′ Enhancer Containing an AP1 Site. J. Biol. Chem..

[192] Neznanov N., Thorey I.S., Ceceña G., Oshima R.G. (1993). Transcriptional Insulation of the Human Keratin 18 Gene in Transgenic Mice. Mol. Cell. Biol..

[193] Oshima R.G., Abrams L., Kulesh D. (1990). Activation of an Intron Enhancer within the Keratin 18 Gene by Expression of C-Fos and c-Jun in Undifferentiated F9 Embryonal Carcinoma Cells. Genes Dev..

[194] Pankov R., Umezawa A., Maki R., Der C.J., Hauser C.A., Oshima R.G. (1994). Oncogene Activation of Human Keratin 18 Transcription via the Ras Signal Transduction Pathway. Proc. Natl. Acad. Sci. USA.

[195] Pankov R., Neznanov N., Umezawa A., Oshima R.G. (1994). AP-1, ETS, and Transcriptional Silencers Regulate Retinoic Acid-Dependent Induction of Keratin 18 in Embryonic Cells. Mol. Cell. Biol..

[196] Neznanov N., Umezawa A., Oshima R.G. (1997). A Regulatory Element within a Coding Exon Modulates Keratin 18 Gene Expression in Transgenic Mice. J. Biol. Chem..

[197] Casanova L., Bravo A., Were F., Ramírez A., Jorcano J.J., Vidal M. (1995). Tissue-Specific and Efficient Expression of the Human Simple Epithelial Keratin 8 Gene in Transgenic Mice. J. Cell Sci..

[198] Soares E., Zhou H. (2018). Master Regulatory Role of P63 in Epidermal Development and Disease. Cell. Mol. Life Sci. CMLS.

[199] Romano R.-A., Smalley K., Magraw C., Serna V.A., Kurita T., Raghavan S., Sinha S. (2012). ΔNp63 Knockout Mice Reveal Its Indispensable Role as a Master Regulator of Epithelial Development and Differentiation. Dev. Camb. Engl..

[200] Bao X., Rubin A.J., Qu K., Zhang J., Giresi P.G., Chang H.Y., Khavari P.A. (2015). A Novel ATAC-Seq Approach Reveals Lineage-Specific Reinforcement of the Open Chromatin Landscape via Cooperation between BAF and P63. Genome Biol..

[201] Kouwenhoven E.N., Oti M., Niehues H., van Heeringen S.J., Schalkwijk J., Stunnenberg H.G., van Bokhoven H., Zhou H. (2015). Transcription Factor P63 Bookmarks and Regulates Dynamic Enhancers during Epidermal Differentiation. EMBO Rep..

[202] Yu X., Singh P.K., Tabrejee S., Sinha S., Buck M.J. (2021). ΔNp63 Is a Pioneer Factor That Binds Inaccessible Chromatin and Elicits Chromatin Remodeling. Epigenetics Chromatin.

[203] Lee A.-Y. (2020). The Role of MicroRNAs in Epidermal Barrier. Int. J. Mol. Sci..

[204] Tiwari N., Meyer-Schaller N., Arnold P., Antoniadis H., Pachkov M., van Nimwegen E., Christofori G. (2013). Klf4 Is a Transcriptional Regulator of Genes Critical for EMT, Including Jnk1 (Mapk8). PLoS ONE.

[205] Mistry D.S., Chen Y., Wang Y., Sen G.L. (2014). SNAI2 Controls the Undifferentiated State of Human Epidermal Progenitor Cells. Stem Cells.

[206] Hudson L.G., Newkirk K.M., Chandler H.L., Choi C., Fossey S.L., Parent A.E., Kusewitt D.F. (2009). Cutaneous Wound Reepithelialization Is Compromised in Mice Lacking Functional Slug (Snai2). J. Dermatol. Sci..

[207] Tripathi M.K., Misra S., Chaudhuri G. (2005). Negative Regulation of the Expressions of Cytokeratins 8 and 19 by SLUG Repressor Protein in Human Breast Cells. Biochem. Biophys. Res. Commun..

[208] Houschyar K.S., Borrelli M.R., Tapking C., Popp D., Puladi B., Ooms M., Chelliah M.P., Rein S., Pförringer D., Thor D. (2020). Molecular Mechanisms of Hair Growth and Regeneration: Current Understanding and Novel Paradigms. Dermatology.

[209] van Genderen C., Okamura R.M., Fariñas I., Quo R.G., Parslow T.G., Bruhn L., Grosschedl R. (1994). Development of Several Organs That Require Inductive Epithelial-Mesenchymal Interactions Is Impaired in LEF-1-Deficient Mice. Genes Dev..

[210] Su Y., Wen J., Zhu J., Xie Z., Liu C., Ma C., Zhang Q., Xu X., Wu X. (2019). Pre-Aggregation of Scalp Progenitor Dermal and Epidermal Stem Cells Activates the WNT Pathway and Promotes Hair Follicle Formation in in Vitro and in Vivo Systems. Stem Cell Res. Ther..

[211] Sharma R., Choi K.-J., Quan M.D., Sharma S., Sankaran B., Park H., LaGrone A., Kim J.J., MacKenzie K.R., Ferreon A.C.M. (2021). Liquid Condensation of Reprogramming Factor KLF4 with DNA Provides a Mechanism for Chromatin Organization. Nat. Commun..

[212] Qu J., Yi G., Zhou H. (2019). P63 Cooperates with CTCF to Modulate Chromatin Architecture in Skin Keratinocytes. Epigenetics Chromatin.

[213] Cremer T., Cremer M. (2010). Chromosome Territories. Cold Spring Harb. Perspect. Biol..

[214] Lieberman-Aiden E., van Berkum N.L., Williams L., Imakaev M., Ragoczy T., Telling A., Amit I., Lajoie B.R., Sabo P.J., Dorschner M.O. (2009). Comprehensive Mapping of Long-Range Interactions Reveals Folding Principles of the Human Genome. Science.

[215] Schoenfelder S., Sexton T., Chakalova L., Cope N.F., Horton A., Andrews S., Kurukuti S., Mitchell J.A., Umlauf D., Dimitrova D.S. (2010). Preferential Associations between Co-Regulated Genes Reveal a Transcriptional Interactome in Erythroid Cells. Nat. Genet..

[216] Monahan K., Horta A., Lomvardas S. (2019). LHX2- and LDB1-Mediated Trans Interactions Regulate Olfactory Receptor Choice. Nature.

[217] Razin S.V., Gavrilov A.A. (2020). The Role of Liquid-Liquid Phase Separation in the Compartmentalization of Cell Nucleus and Spatial Genome Organization. Biochem. Biokhimiia.

[218] Lu H., Yu D., Hansen A.S., Ganguly S., Liu R., Heckert A., Darzacq X., Zhou Q. (2018). Phase-Separation Mechanism for C-Terminal Hyperphosphorylation of RNA Polymerase II. Nature.

[219] Hansen J.C., Maeshima K., Hendzel M.J. (2021). The Solid and Liquid States of Chromatin. Epigenetics Chromatin.

[220] Dixon J.R., Selvaraj S., Yue F., Kim A., Li Y., Shen Y., Hu M., Liu J.S., Ren B. (2012). Topological Domains in Mammalian Genomes Identified by Analysis of Chromatin Interactions. Nature.

[221] Sexton T., Yaffe E., Kenigsberg E., Bantignies F., Leblanc B., Hoichman M., Parrinello H., Tanay A., Cavalli G. (2012). Three-Dimensional Folding and Functional Organization Principles of the Drosophila Genome. Cell.

[222] Brandão H.B., Paul P., van den Berg A.A., Rudner D.Z., Wang X., Mirny L.A. (2019). RNA Polymerases as Moving Barriers to Condensin Loop Extrusion. Proc. Natl. Acad. Sci. USA.

[223] Rao S.S.P., Huntley M.H., Durand N.C., Stamenova E.K., Bochkov I.D., Robinson J.T., Sanborn A.L., Machol I., Omer A.D., Lander E.S. (2014). A 3D Map of the Human Genome at Kilobase Resolution Reveals Principles of Chromatin Looping. Cell.

[224] Nora E.P., Goloborodko A., Valton A.-L., Gibcus J.H., Uebersohn A., Abdennur N., Dekker J., Mirny L.A., Bruneau B.G. (2017). Targeted Degradation of CTCF Decouples Local Insulation of Chromosome Domains from Genomic Compartmentalization. Cell.

[225] Rao S.S.P., Huang S.-C., Glenn St Hilaire B., Engreitz J.M., Perez E.M., Kieffer-Kwon K.-R., Sanborn A.L., Johnstone S.E., Bascom G.D., Bochkov I.D. (2017). Cohesin Loss Eliminates All Loop Domains. Cell.

[226] Dixon J.R., Jung I., Selvaraj S., Shen Y., Antosiewicz-Bourget J.E., Lee A.Y., Ye Z., Kim A., Rajagopal N., Xie W. (2015). Chromatin Architecture Reorganization during Stem Cell Differentiation. Nature.

[227] Pope B.D., Ryba T., Dileep V., Yue F., Wu W., Denas O., Vera D.L., Wang Y., Hansen R.S., Canfield T.K. (2014). Topologically Associating Domains Are Stable Units of Replication-Timing Regulation. Nature.

[228] Nora E.P., Lajoie B.R., Schulz E.G., Giorgetti L., Okamoto I., Servant N., Piolot T., van Berkum N.L., Meisig J., Sedat J. (2012). Spatial Partitioning of the Regulatory Landscape of the X-Inactivation Centre. Nature.

[229] Guo Y., Xu Q., Canzio D., Shou J., Li J., Gorkin D.U., Jung I., Wu H., Zhai Y., Tang Y. (2015). CRISPR Inversion of CTCF Sites Alters Genome Topology and Enhancer/Promoter Function. Cell.

[230] Sehgal N., Seifert B., Ding H., Chen Z., Stojkovic B., Bhattacharya S., Xu J., Berezney R. (2016). Reorganization of the Interchromosomal Network during Keratinocyte Differentiation. Chromosoma.

[231] Cavazza A., Miccio A., Romano O., Petiti L., Malagoli Tagliazucchi G., Peano C., Severgnini M., Rizzi E., De Bellis G., Bicciato S. (2016). Dynamic Transcriptional and Epigenetic Regulation of Human Epidermal Keratinocyte Differentiation. Stem Cell Rep..

[232] Rubin A.J., Barajas B.C., Furlan-Magaril M., Lopez-Pajares V., Mumbach M.R., Howard I., Kim D.S., Boxer L.D., Cairns J., Spivakov M. (2017). Lineage-Specific Dynamic and Pre-Established Enhancer-Promoter Contacts Cooperate in Terminal Differentiation. Nat. Genet..

[233] Poterlowicz K., Yarker J.L., Malashchuk I., Lajoie B.R., Mardaryev A.N., Gdula M.R., Sharov A.A., Kohwi-Shigematsu T., Botchkarev V.A., Fessing M.Y. (2017). 5C Analysis of the Epidermal Differentiation Complex Locus Reveals Distinct Chromatin Interaction Networks between Gene-Rich and Gene-Poor TADs in Skin Epithelial Cells. PLoS Genet..

[234] Chen G.-D., Fatima I., Xu Q., Rozhkova E., Fessing M.Y., Mardaryev A.N., Sharov A.A., Xu G.-L., Botchkarev V.A. (2023). DNA Dioxygenases Tet2/3 Regulate Gene Promoter Accessibility and Chromatin Topology in Lineage-Specific Loci to Control Epithelial Differentiation. Sci. Adv..

[235] Liu X.S., Wu H., Ji X., Stelzer Y., Wu X., Czauderna S., Shu J., Dadon D., Young R.A., Jaenisch R. (2016). Editing DNA Methylation in the Mammalian Genome. Cell.

[236] Liang Y.-C., Wu P., Lin G.-W., Chen C.-K., Yeh C.-Y., Tsai S., Yan J., Jiang T.-X., Lai Y.-C., Huang D. (2020). Folding Keratin Gene Clusters during Skin Regional Specification. Dev. Cell.

[237] Abaci H.E., Coffman A., Doucet Y., Chen J., Jacków J., Wang E., Guo Z., Shin J.U., Jahoda C.A., Christiano A.M. (2018). Tissue Engineering of Human Hair Follicles Using a Biomimetic Developmental Approach. Nat. Commun..

[238] Ji S., Zhu Z., Sun X., Fu X. (2021). Functional Hair Follicle Regeneration: An Updated Review. Signal Transduct. Target. Ther..

[239] Yamaguchi Y., Itami S., Tarutani M., Hosokawa K., Miura H., Yoshikawa K. (1999). Regulation of Keratin 9 in Nonpalmoplantar Keratinocytes by Palmoplantar Fibroblasts Through Epithelial–Mesenchymal Interactions. J. Investig. Dermatol..

[240] Yang X., Moldovan N., Zhao Q., Mi S., Zhou Z., Chen D., Gao Z., Tong D., Dou Z. (2008). Reconstruction of Damaged Cornea by Autologous Transplantation of Epidermal Adult Stem Cells. Mol. Vis..

[241] Rogovaya O.S., Fayzulin A.K., Vasiliev A.V., Kononov A.V., Terskikh V.V. (2015). Reconstruction of Rabbit Urethral Epithelium with Skin Keratinocytes. Acta Nat..

[242] Yao S., Huang H.-Y., Han X., Ye Y., Qin Z., Zhao G., Li F., Hu G., Hu L., Ji H. (2019). Keratin 14-High Subpopulation Mediates Lung Cancer Metastasis Potentially through Gkn1 Upregulation. Oncogene.

[243] Ogunnigbagbe O., Bunick C.G., Kaur K. (2022). Keratin 1 as a Cell-Surface Receptor in Cancer. Biochim. Biophys. Acta Rev. Cancer.

[244] Hu W.-Y., Hu D.-P., Xie L., Nonn L., Lu R., Abern M., Shioda T., Prins G.S. (2021). Keratin Profiling by Single-Cell RNA-Sequencing Identifies Human Prostate Stem Cell Lineage Hierarchy and Cancer Stem-Like Cells. Int. J. Mol. Sci..

[245] Zhang F., Wang G., Yan W., Jiang H. (2022). MiR-4268 Suppresses Gastric Cancer Genesis through Inhibiting Keratin 80. Cell Cycle Georget. Tex.

[246] Baraks G., Tseng R., Pan C.-H., Kasliwal S., Leiton C.V., Shroyer K.R., Escobar-Hoyos L.F. (2022). Dissecting the Oncogenic Roles of Keratin 17 in the Hallmarks of Cancer. Cancer Res..

[247] Roa-Peña L., Babu S., Leiton C.V., Wu M., Taboada S., Akalin A., Buscaglia J., Escobar-Hoyos L.F., Shroyer K.R. (2021). Keratin 17 Testing in Pancreatic Cancer Needle Aspiration Biopsies Predicts Survival. Cancer Cytopathol..

[248] Wang W., Lozar T., Golfinos A.E., Lee D., Gronski E., Ward-Shaw E., Hayes M., Bruce J.Y., Kimple R.J., Hu R. (2022). Stress Keratin 17 Expression in Head and Neck Cancer Contributes to Immune Evasion and Resistance to Immune-Checkpoint Blockade. Clin. Cancer Res. Off. J. Am. Assoc. Cancer Res..

[249] Ouyang S., Kang W.-M. (2022). Research Advances in the Role of Keratins in Gastrointestinal Cancer. Chin. Med. Sci. J..

[250] Sequeira I., Neves J.F., Carrero D., Peng Q., Palasz N., Liakath-Ali K., Lord G.M., Morgan P.R., Lombardi G., Watt F.M. (2018). Immunomodulatory Role of Keratin 76 in Oral and Gastric Cancer. Nat. Commun..

[251] Roa-Peña L., Leiton C.V., Babu S., Pan C.-H., Vanner E.A., Akalin A., Bandovic J., Moffitt R.A., Shroyer K.R., Escobar-Hoyos L.F. (2019). Keratin 17 Identifies the Most Lethal Molecular Subtype of Pancreatic Cancer. Sci. Rep..

[252] Zhu R.-J., Zhou J., Liang P.-Q., Xiang X.-X., Ran J., Xie T.-A., Guo X.-G. (2022). Accuracy of Cytokeratin 19 Fragment in the Diagnosis of Bladder Cancer. Biomark. Med..

[253] Hung C.-S., Wang Y.-C., Guo J.-W., Yang R.-N., Lee C.-L., Shen M.-H., Huang C.-C., Huang C.-J., Yang J.-Y., Liu C.-Y. (2020). Expression Pattern of Placenta Specific 8 and Keratin 20 in Different Types of Gastrointestinal Cancer. Mol. Med. Rep..

[254] Kim J., Villadsen R. (2020). The Expression Pattern of Epidermal Differentiation Marker Keratin 10 in the Normal Human Breast and Breast Cancer Cells. J. Histochem. Cytochem. Off. J. Histochem. Soc..

[255] Rao X., Wang J., Song H.M., Deng B., Li J.G. (2020). KRT15 Overexpression Predicts Poor Prognosis in Colorectal Cancer. Neoplasma.

[256] Bai J.D.K., Babu S., Roa-Peña L., Hou W., Akalin A., Escobar-Hoyos L.F., Shroyer K.R. (2019). Keratin 17 Is a Negative Prognostic Biomarker in High-Grade Endometrial Carcinomas. Hum. Pathol..

[257] Lee H.E., Torbenson M.S., Wu T.-T., Chandan V.S. (2020). Aberrant Keratin Expression Is Common in Primary Hepatic Malignant Vascular Tumors: A Potential Diagnostic Pitfall. Ann. Diagn. Pathol..

[258] Werner S., Keller L., Pantel K. (2020). Epithelial Keratins: Biology and Implications as Diagnostic Markers for Liquid Biopsies. Mol. Aspects Med..

[259] Zhang Z., Tu K., Liu F., Liang M., Yu K., Wang Y., Luo Y., Yang B., Qin Y., He D. (2020). FoxM1 Promotes the Migration of Ovarian Cancer Cell through KRT5 and KRT7. Gene.

[260] Ji R., Ji Y., Ma L., Ge S., Chen J., Wu S., Huang T., Sheng Y., Wang L., Yi N. (2021). Keratin 17 Upregulation Promotes Cell Metastasis and Angiogenesis in Colon Adenocarcinoma. Bioengineered.

[261] Liu L., Sun L., Zheng J., Cui L. (2021). Berberine Modulates Keratin 17 to Inhibit Cervical Cancer Cell Viability and Metastasis. J. Recept. Signal Transduct. Res..

[262] Yang B., Zhang W., Zhang M., Wang X., Peng S., Zhang R. (2020). KRT6A Promotes EMT and Cancer Stem Cell Transformation in Lung Adenocarcinoma. Technol. Cancer Res. Treat..

[263] Ren M., Gao Y., Chen Q., Zhao H., Zhao X., Yue W. (2020). The Overexpression of Keratin 23 Promotes Migration of Ovarian Cancer via Epithelial-Mesenchymal Transition. BioMed Res. Int..

[264] Tsai F.-J., Lai M.-T., Cheng J., Chao S.C.-C., Korla P.K., Chen H.-J., Lin C.-M., Tsai M.-H., Hua C.-H., Jan C.-I. (2019). Novel K6-K14 Keratin Fusion Enhances Cancer Stemness and Aggressiveness in Oral Squamous Cell Carcinoma. Oncogene.

[265] Hu H.-B., Yang X.-P., Zhou P.-X., Yang X.-A., Yin B. (2020). High Expression of Keratin 6C Is Associated with Poor Prognosis and Accelerates Cancer Proliferation and Migration by Modulating Epithelial-Mesenchymal Transition in Lung Adenocarcinoma. Genes Genom..

[266] Lim S.-C., Parajuli K.R., Han S.I. (2019). Keratin 6, Induced by Chronic Cisplatin Exposure, Confers Chemoresistance in Human Gastric Carcinoma Cells. Oncol. Rep..

[267] Liu Z., Yu S., Ye S., Shen Z., Gao L., Han Z., Zhang P., Luo F., Chen S., Kang M. (2020). Keratin 17 Activates AKT Signalling and Induces Epithelial-Mesenchymal Transition in Oesophageal Squamous Cell Carcinoma. J. Proteom..

[268] Li J., Chen Q., Deng Z., Chen X., Liu H., Tao Y., Wang X., Lin S., Liu N. (2019). KRT17 Confers Paclitaxel-Induced Resistance and Migration to Cervical Cancer Cells. Life Sci..

[269] Liu J., Liu L., Cao L., Wen Q. (2018). Keratin 17 Promotes Lung Adenocarcinoma Progression by Enhancing Cell Proliferation and Invasion. Med. Sci. Monit. Int. Med. J. Exp. Clin. Res..

[270] Zieman A.G., Poll B.G., Ma J., Coulombe P.A. (2019). Altered Keratinocyte Differentiation Is an Early Driver of Keratin Mutation-Based Palmoplantar Keratoderma. Hum. Mol. Genet..

[271] Spörrer M., Prochnicki A., Tölle R.C., Nyström A., Esser P.R., Homberg M., Athanasiou I., Zingkou E., Schilling A., Gerum R. (2019). Treatment of Keratinocytes with 4-Phenylbutyrate in Epidermolysis Bullosa: Lessons for Therapies in Keratin Disorders. eBioMedicine.

[272] Westin M., Rekabdar E., Blomstrand L., Klintberg P., Jontell M., Robledo-Sierra J. (2018). Mutations in the Genes for Keratin-4 and Keratin-13 in Swedish Patients with White Sponge Nevus. J. Oral Pathol. Med. Off. Publ. Int. Assoc. Oral Pathol. Am. Acad. Oral Pathol..

[273] Mathews J., Hansen C.D., Chandrashekar L. (2021). Homozygous Dominant Missense Mutation in Keratin 6b Leading to Severe Pachyonychia Congenita. Clin. Exp. Dermatol..

[274] Komine M. (2018). Regulation of Expression of Keratins and Their Pathogenic Roles in Keratinopathies.

[275] Logli E., Marzuolo E., D’Agostino M., Conti L.A., Lena A.M., Diociaiuti A., Dellambra E., Has C., Cianfanelli V., Zambruno G. (2022). Proteasome-Mediated Degradation of Keratins 7, 8, 17 and 18 by Mutant KLHL24 in a Foetal Keratinocyte Model: Novel Insight in Congenital Skin Defects and Fragility of Epidermolysis Bullosa Simplex with Cardiomyopathy. Hum. Mol. Genet..

[276] Pan C.-Y., Chou C.-C. (2021). Molecular Origin of the Effects of Mutation on the Structure and Mechanical Properties of Human Epithelial Keratin K5/K14. J. Mech. Behav. Biomed. Mater..

[277] Chen F., Yao L., Zhang X., Gu Y., Yu H., Yao Z., Zhang J., Li M. (2021). Damaged Keratin Filament Network Caused by KRT5 Mutations in Localized Recessive Epidermolysis Bullosa Simplex. Front. Genet..

[278] Fujiwara S., Deguchi S., Magin T.M. (2020). Disease-Associated Keratin Mutations Reduce Traction Forces and Compromise Adhesion and Collective Migration. J. Cell Sci..

[279] Pânzaru M.-C., Caba L., Florea L., Braha E.E., Gorduza E.V. (2022). Epidermolysis Bullosa—A Different Genetic Approach in Correlation with Genetic Heterogeneity. Diagnostics.

[280] Evtushenko N.A., Beilin A.K., Kosykh A.V., Vorotelyak E.A., Gurskaya N.G. (2021). Keratins as an Inflammation Trigger Point in Epidermolysis Bullosa Simplex. Int. J. Mol. Sci..

[281] Spaunhurst K.M., Hogendorf A.M., Smith F.J.D., Lingala B., Schwartz M.E., Cywinska-Bernas A., Zeman K.J., Tang J.Y. (2012). Pachyonychia Congenita Patients with Mutations in KRT6A Have More Extensive Disease Compared with Patients Who Have Mutations in KRT16. Br. J. Dermatol..

[282] Wu T.T., Eldirany S.A., Bunick C.G., Teng J.M.C. (2021). Genotype-Structurotype-Phenotype Correlations in Patients with Pachyonychia Congenita. J. Investig. Dermatol..

[283] Vodo D., Sarig O., Peled A., Samuelov L., Malchin N., Grafi-Cohen M., Sprecher E. (2018). Recessive Epidermolytic Ichthyosis Results from Loss of Keratin 10 Expression, Regardless of the Mutation Location. Clin. Exp. Dermatol..

[284] Smith F.J.D., Kreuser-Genis I.M., Jury C.S., Wilson N.J., Terron-Kwiatowski A., Zamiri M. (2019). Novel and Recurrent Mutations in Keratin 1 Cause Epidermolytic Ichthyosis and Palmoplantar Keratoderma. Clin. Exp. Dermatol..

[285] Suzuki Y., Takeichi T., Tanahashi K., Muro Y., Suga Y., Ogi T., Akiyama M. (2022). Deep Phenotyping of Superficial Epidermolytic Ichthyosis Due to a Recurrent Mutation in KRT2. Int. J. Mol. Sci..

[286] Ye J., Wu Y., Li M., Gong X., Zhong B. (2020). Keratin 8 Mutations Were Associated With Susceptibility to Chronic Hepatitis B and Related Progression. J. Infect. Dis..

[287] Yi H., Yoon H.-N., Kim S., Ku N.-O. (2018). The Role of Keratins in the Digestive System: Lessons from Transgenic Mouse Models. Histochem. Cell Biol..

[288] Luan X.-R., Chen X.-L., Tang Y.-X., Zhang J.-Y., Gao X., Ke H.-P., Lin Z.-Y., Zhang X.-N. (2018). CRISPR/Cas9-Mediated Treatment Ameliorates the Phenotype of the Epidermolytic Palmoplantar Keratoderma-like Mouse. Mol. Ther.-Nucleic Acids.

[289] Koller U., Bauer J.W. (2021). Gene Replacement Therapies for Genodermatoses: A Status Quo. Front. Genet..

[290] Claringbould A., Zaugg J.B. (2021). Enhancers in Disease: Molecular Basis and Emerging Treatment Strategies. Trends Mol. Med..

[291] Zhang G., Shi J., Zhu S., Lan Y., Xu L., Yuan H., Liao G., Liu X., Zhang Y., Xiao Y. (2018). DiseaseEnhancer: A Resource of Human Disease-Associated Enhancer Catalog. Nucleic Acids Res..

[292] Karnuta J.M., Scacheri P.C. (2018). Enhancers: Bridging the Gap between Gene Control and Human Disease. Hum. Mol. Genet..

[293] Kircher M., Xiong C., Martin B., Schubach M., Inoue F., Bell R.J.A., Costello J.F., Shendure J., Ahituv N. (2019). Saturation Mutagenesis of Twenty Disease-Associated Regulatory Elements at Single Base-Pair Resolution. Nat. Commun..

[294] Maurya S.S. (2021). Role of Enhancers in Development and Diseases. Epigenomes.

[295] Tena J.J., Santos-Pereira J.M. (2021). Topologically Associating Domains and Regulatory Landscapes in Development, Evolution and Disease. Front. Cell Dev. Biol..

[296] Luchnik A.N. (2021). Chromosome Instability Induced by Mutations in TAD Anchors Leads to Tumors. Genome Instab. Dis..

[297] Bruneau B.G., Nora E.P. (2018). Chromatin Domains Go on Repeat in Disease. Cell.

